# Supplementary material for: High-performance flexible p-type Ce-filled Fe3CoSb12 skutterudite thin film for medium-to-high-temperature applications
Source: Nat Commun. 2024 May 18;15:4242. doi: 10.1038/s41467-024-48677-4 (PMC11102547; doi:10.1038/s41467-024-48677-4)
Supplement: Supplementary file 1 — Supplementary Information [file 41467_2024_48677_MOESM1_ESM.pdf]

Supporting Information

High-performance flexible p-type Ce-filled Fe_3CoSb_{12} skutterudite thin film for medium-to-high-temperature applications

Dou Li,[1,a] Xiao-Lei Shi,[2,a] Jiaxi Zhu,[1] Tianyi Cao,[2] Xiao Ma,[1] Meng Li,[2] Zhuokun Han,[3] Zhenyu Feng,[1] Yixing Chen,[1] Jianyuan Wang,[3] Wei-Di Liu,[2] Hong Zhong,[1,] Shuangming Li,[1,*] Zhi-Gang Chen[2,*]*

[1] State Key Laboratory of Solidification Processing, Northwestern Polytechnical University, Xi'an 710072, P. R. China.

[2] School of Chemistry and Physics, ARC Research Hub in Zero-emission Power Generation for Carbon Neutrality, and Centre for Materials Science, Queensland University of Technology, Brisbane, Queensland 4000, Australia;

[3] MOE Key Laboratory of Material Physics and Chemistry Under Extraordinary Conditions, School of Physical Science and Technology, Northwestern Polytechnical University, Xi'an, 710072, P. R. China.

[a] These authors contributed equally: Dou Li, Xiao-Lei Shi.

Corresponding Author:

* Hong Zhong: zhonghong123@nwpu.edu.cn;

* Shuangming Li: lsm@nwpu.edu.cn;

* Zhi-Gang Chen: zhigang.chen@qut.edu.au.

Calculation Details

First-principles calculations: First-principles calculations were performed based on density-functional theory (DFT) with the all-electron projected augmented wave (PAW) method, as implemented in the Vienna Ab initio Simulation Package (VASP) [1-6]. Semi-local generalized gradient approximation (GGA) with the fully relativistic Perdew-Burke-Ernzerhof (PBE) exchange-correlation functional was employed [7]. The valence wave functions were expanded on a plan-wave basis with a cut-off energy of 450 eV. All atoms were allowed to relax in their geometric optimizations, with a convergence criterion of 1×10^{-7} eV per electron and 1×10^{-3} eV·Å^{-1} per unit cell. The Brillouin zone was sampled by a Monkhorst-Pack **k**-mesh spanning less than 0.06/Å^3 for structural relaxation, and a denser **k**-mesh spanning less than 0.03/Å^3 for calculating static self-consistency and density-of-state (DOS). The electron band structures were calculated along the line-mode **k**-path based on Brillouin path features indicated by the AFLOW framework [8]. To precisely predict bandgap, the Hubbard U model was considered; The on-site coulombic (U) and the exchange (J) terms were determined using a linear corresponding method.

SPB modeling: There are [9-12]:

$$S(\eta) = \frac{k_B}{e} \cdot \left[\frac{\left(r+\frac{5}{2}\right)\cdot F_{r+\frac{3}{2}}(\eta)}{\left(r+\frac{3}{2}\right)\cdot F_{r+\frac{1}{2}}(\eta)} - \eta \right] \tag{S1-1}$$

$$p = \frac{1}{e\cdot R_H} = \frac{(2m^*\cdot k_B T)^{\frac{3}{2}}}{3\pi^2\hbar^3} \cdot \frac{\left(r+\frac{3}{2}\right)^2\cdot F^2_{r+\frac{1}{2}}(\eta)}{(2r+\frac{3}{2})\cdot F_{2r+\frac{1}{2}}(\eta)} \tag{S1-2}$$

$$\mu = \left[\frac{e\pi\hbar^4}{\sqrt{2}(k_B T)^{\frac{3}{2}}} \frac{C_l}{E_{def}{}^2(m^*)^{\frac{5}{2}}} \right] \frac{(2r+\frac{3}{2})\cdot F_{2r+\frac{1}{2}}(\eta)}{\left(r+\frac{3}{2}\right)^2\cdot F_{r+\frac{1}{2}}(\eta)} \tag{S1-3}$$

$$L = (\frac{k_B}{e})^2 \cdot \left\{ \frac{\left(r+\frac{7}{2}\right)\cdot F_{r+\frac{5}{2}}(\eta)}{\left(r+\frac{3}{2}\right)\cdot F_{r+\frac{1}{2}}(\eta)} - \left[\frac{\left(r+\frac{5}{2}\right)\cdot F_{r+\frac{3}{2}}(\eta)}{\left(r+\frac{3}{2}\right)\cdot F_{r+\frac{1}{2}}(\eta)} \right]^2 \right\} \tag{S1-4}$$

where η is the reduced Fermi level, k_B is the Boltzmann constant, r is the carrier scattering factor (r = −1/2 for acoustic phonon scattering), R_H is the Hall coefficient, C_l is the elastic constant for longitudinal vibrations, and E_{def} is the deformation potential coefficient, respectively. There is [9-12]:

$$C_l = v_L^2 \cdot d \tag{S1-5}$$

The longitudinal velocity v_L is derived from the reference [13-15]. $F_i(\eta)$ is the Fermi integral, expressed as [9-12]:

$$F_i(\eta) = \int_0^\infty \frac{x^i}{1+e^{(x-\eta)}} dx \tag{S1-6}$$

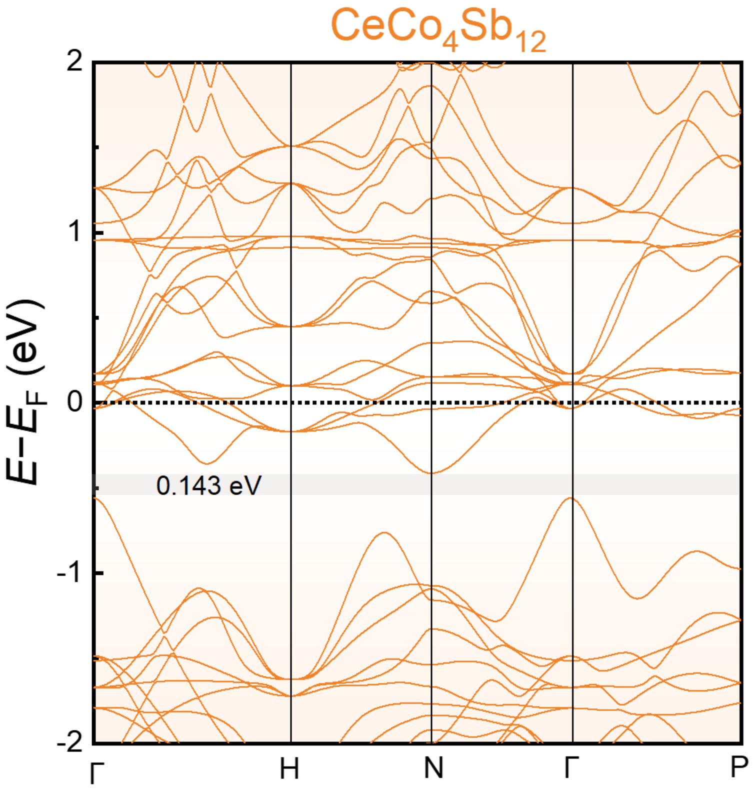

Supplementary Fig. 1 Calculated band structure of $CeCo_4Sb_{12}$.

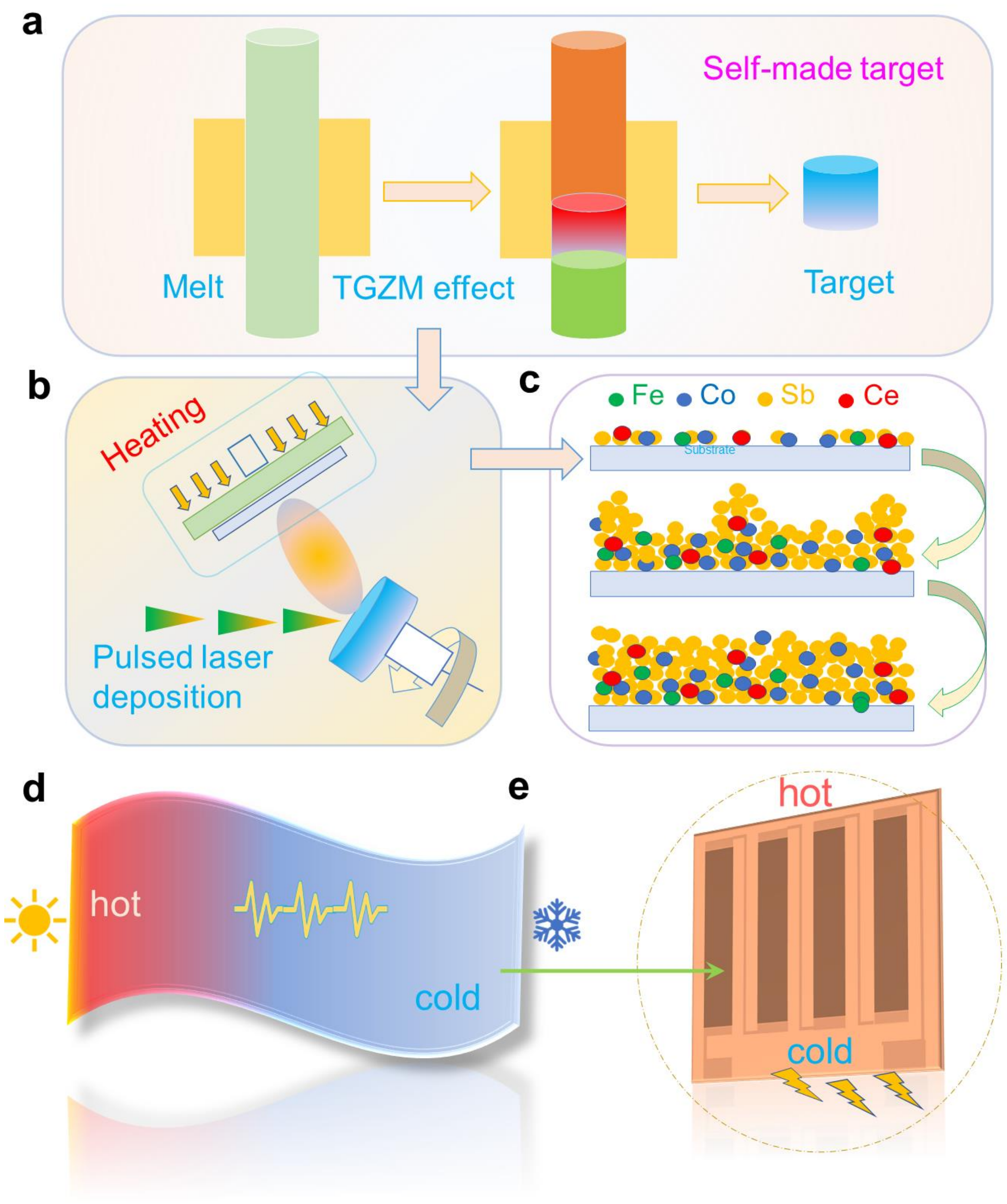

Supplementary Fig. 2 Schematic diagram of fabrication process of thin-film thermoelectric materials and devices. (**a**) Self-made $Ce_xFe_3CoSb_{12}$-based pulse laser deposition (PLD) target (x = 0.25, 0.50, 0.75, 1.25, and 1.50) prepared based on the temperature gradient zone melting (TGZM) effect [16]. (**b**) Illustration of the PLD process. (**c**) Illustration of the growth process of $Ce_xFe_3CoSb_{12}$-based thin films. (**d**) Schematic diagram of the prepared flexible $Ce_xFe_3CoSb_{12}$-based thermoelectric thin films. (**e**) Schematic diagram of the thermoelectric device made from the flexible $Ce_xFe_3CoSb_{12}$-based thermoelectric thin films.

Shenzhen Runsea Electronic Co.,Ltd
深圳市润海电子有限公司

PI Film Test Report
产品物性报告单(PI薄膜)

序号 No.	指 标 名 称 Test content name		单位 Unit	指标值 Tolerance Range	检测结果 Test result
1	Surface 外观		-	Amber polyimide Film ,surface smooth, no wrinkles and bubbles, neat edges without damage.	Pass
2	Film roll and core膜卷与管芯		mm	As customed 客户要求	— / —
3	Thickness 厚度		um	25/50/75/100/125/150/175/200/225/250	25/50/75/100/125/150/175/200/225/250
	Thickness Tolerance 厚度允许偏差			±5	—
4	Joint接头数		each1000m	≤2	0
	length		m	400	—
5	Width 薄膜宽度		MM	520	520
	Width Tolerance 宽度允许偏差			±5	-
6	Tensile Strength 拉伸强度	MD纵向	Mpa	≥135	139
		TD横向		≥115	118
7	Elongation 延伸率	MD纵向	%	≥35	55
		TD横向			70
8	Insulation Strength 绝缘强度	平均值 Average	MV/m	≥135	135
		个别值 Individual		≥100	110
9	Sruface Resistivity表面电阻率, 200℃		Ω	≥1.0*10^{13}	1.1*10^{13}
10	Shrinkage收缩率, 250℃±5	MD纵向	‰	≤1.0	0.3
		TD横向		≤0.9	0.12
11	Breakdown voltage击穿电压	25um厚	KV	≥3KV	3KV
		50/75/100/125/150/175/200/225/250um厚	KV	≥5KV	5KV
12	Temperature resistance耐温	25/50um厚	℃	250	250
		75/100/125/150/175/200/225/250um厚	℃	350	350
13	Volume Resistivity体积电阻, 200℃		Ω m	≥1.0*10^{13}	1.0*10^{13}

Supplementary Fig. 3 Detailed information on the polyimide (PI) films used as the flexible supporting substrates for all $Ce_xFe_3CoSb_{12}$-based thin films (x = 0.25, 0.50, 0.75, 1.25, and 1.50) in this work.

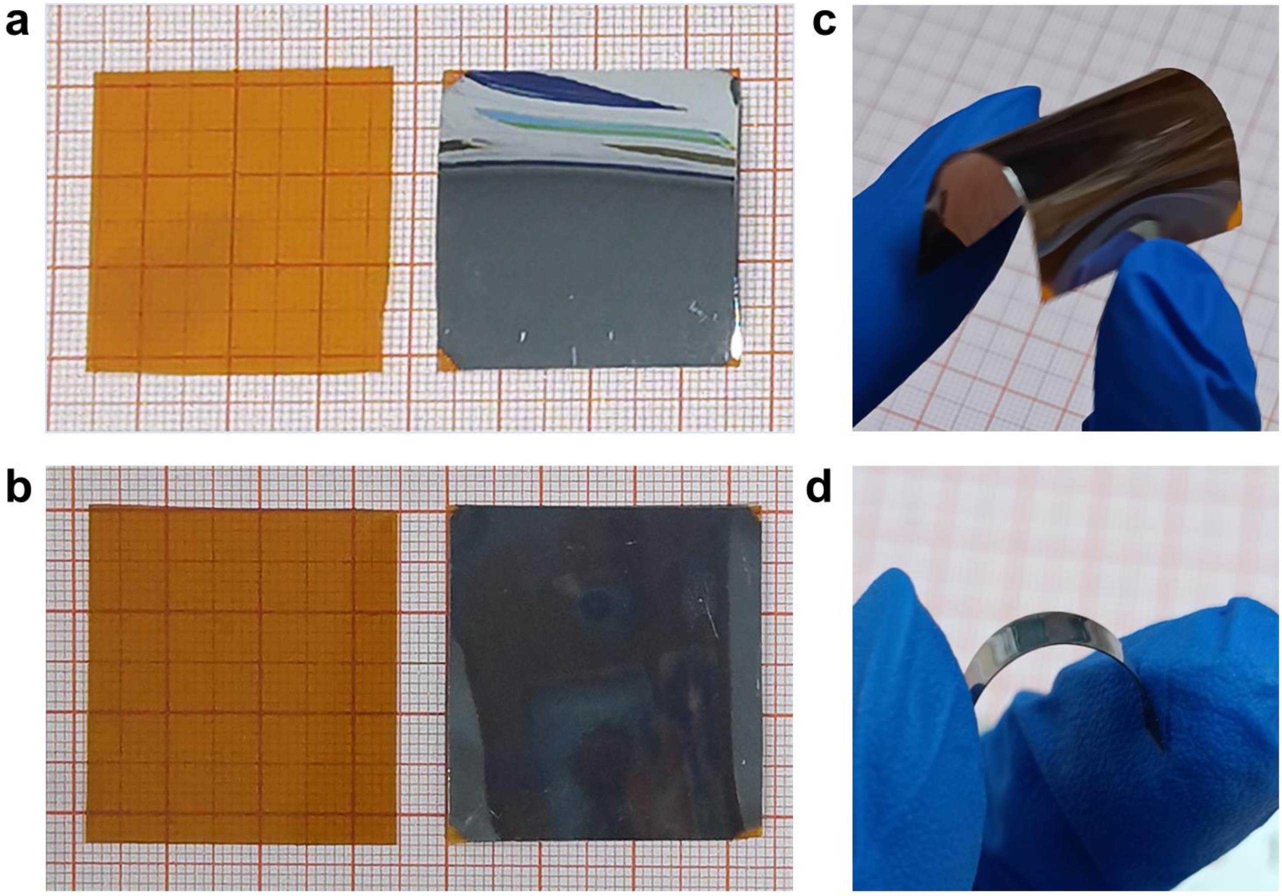

Supplementary Fig. 4 Photos of skutterudite thin films before and after deposition and the bending state of the films. (**a-b**) Photographs of PI substrates and p-type $Ce_{1.25}Fe_3CoSb_{12}$ skutterudite thin films on the substrates from top views. (**c-d**) Schematic diagrams depicting films of various sizes that can be bent.

a

b

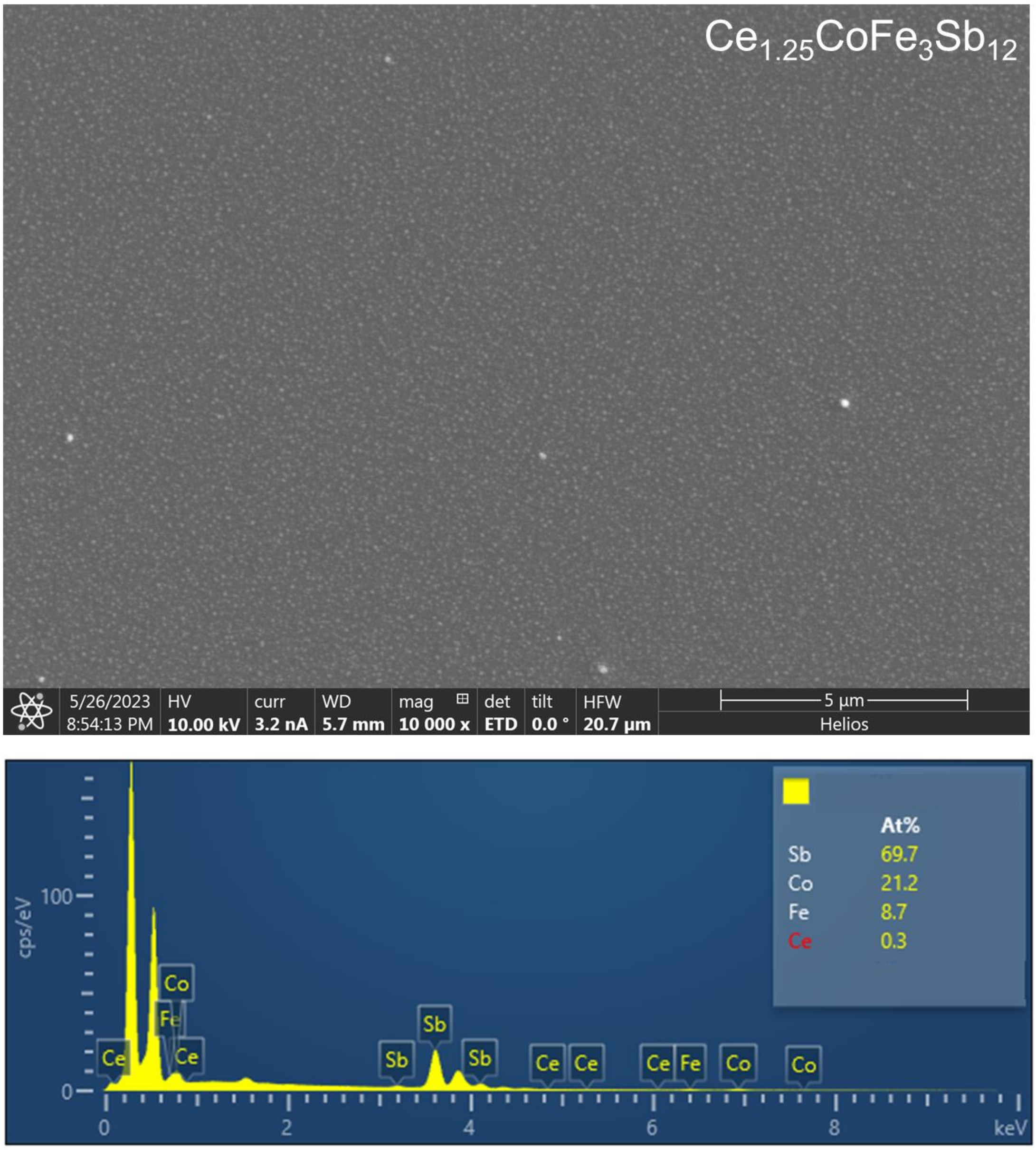

Supplementary Fig. 5 The surface morphology and composition of $Ce_{1.25}Fe_3CoSb_{12}$ skutterudite thin film. (**a**) Enlarged version of the original scanning electron microscopy (SEM) image of $Ce_{1.25}Fe_3CoSb_{12}$ skutterudite thin film from a top view. (**b**) Corresponding energy dispersive X-ray spectrometry (EDS) result.

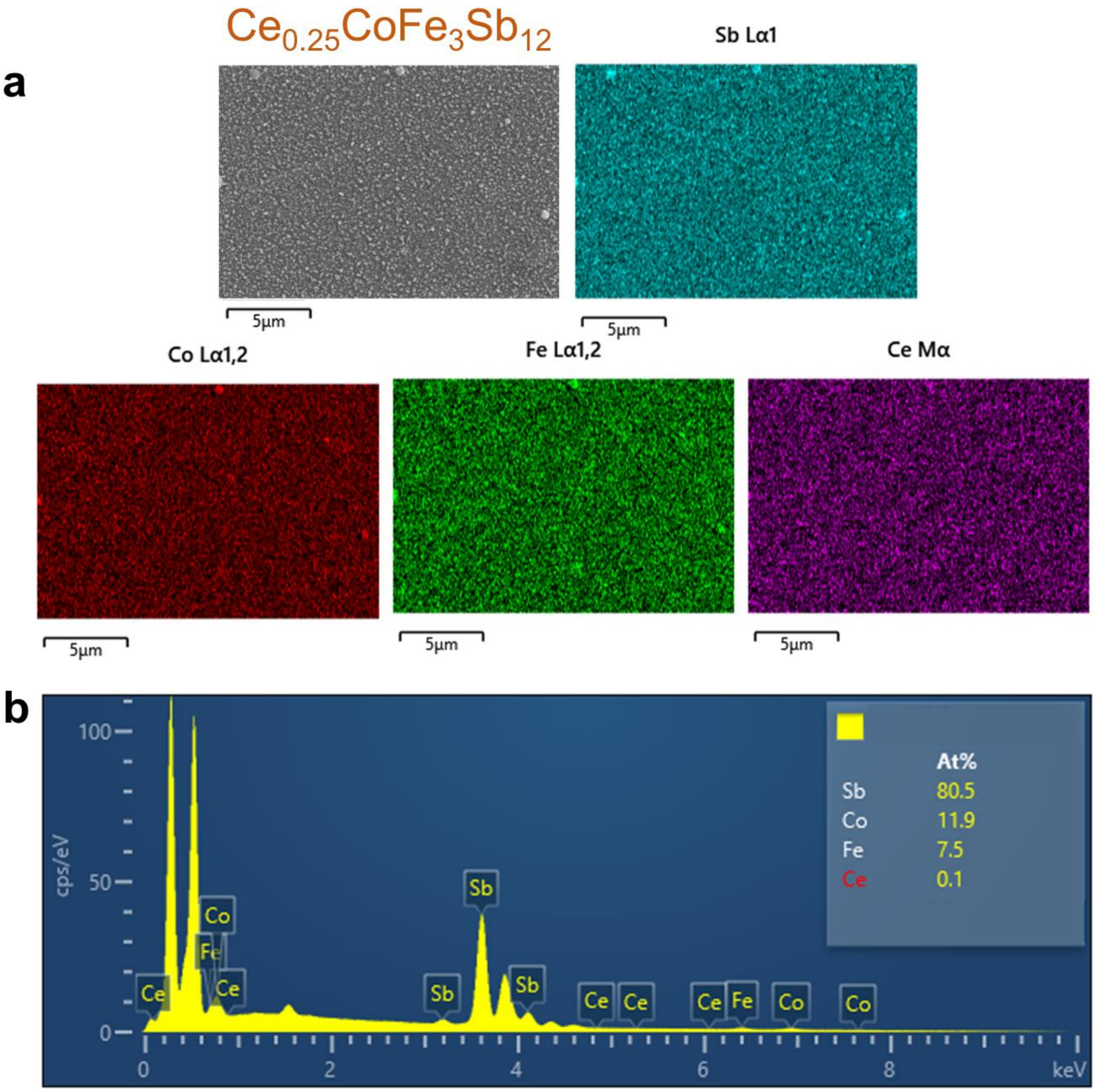

Supplementary Fig. 6 The surface morphology and composition of $Ce_{0.25}Fe_3CoSb_{12}$ skutterudite thin film. (**a**) SEM image of $Ce_{0.25}Fe_3CoSb_{12}$ skutterudite thin film from a top view and corresponding EDS maps for Sb, Co, Fe, and Ce. (**b**) Corresponding EDS composition results taken from the EDS maps.

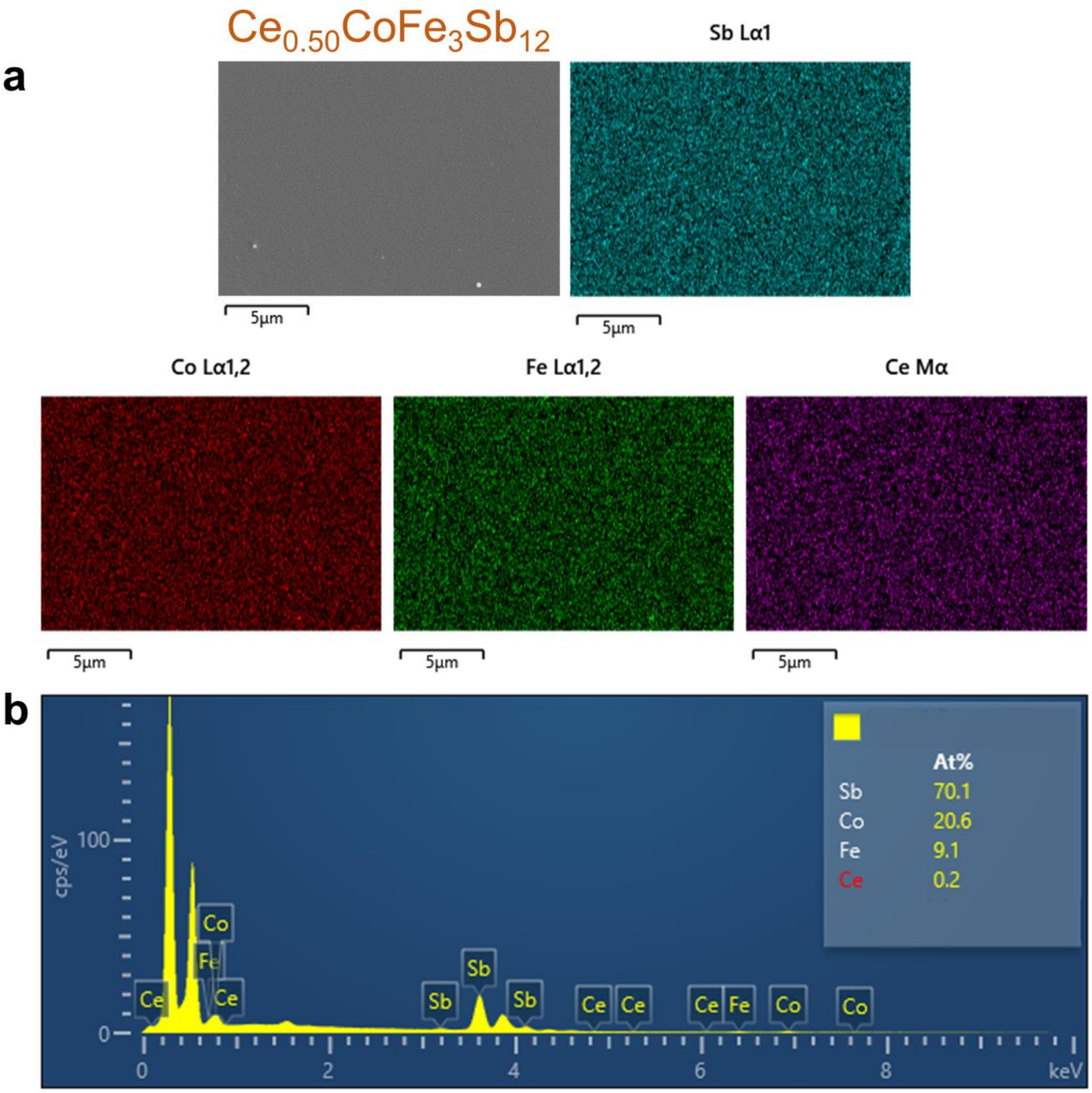

Supplementary Fig. 7 The surface morphology and composition of $Ce_{0.50}Fe_3CoSb_{12}$ skutterudite thin film. (**a**) SEM image of $Ce_{0.50}Fe_3CoSb_{12}$ skutterudite thin film from a top view and corresponding EDS maps for Sb, Co, Fe, and Ce. (**b**) Corresponding EDS composition results taken from the EDS maps.

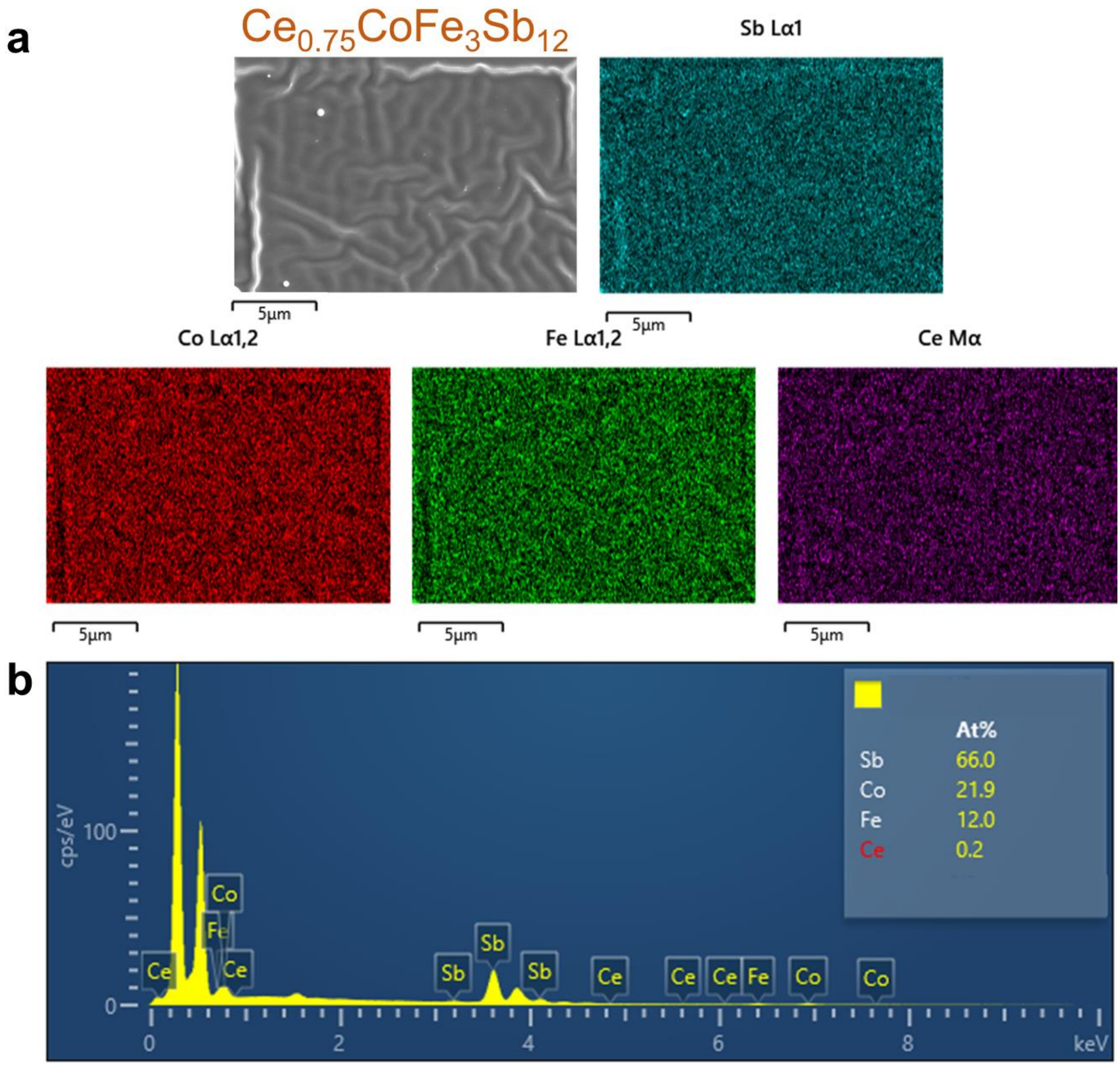

Supplementary Fig. 8 The surface morphology and composition of $Ce_{0.75}Fe_3CoSb_{12}$ skutterudite thin film. (**a**) SEM image of $Ce_{0.75}Fe_3CoSb_{12}$ skutterudite thin film from a top view and corresponding EDS maps for Sb, Co, Fe, and Ce. (**b**) Corresponding EDS composition results taken from the EDS maps.

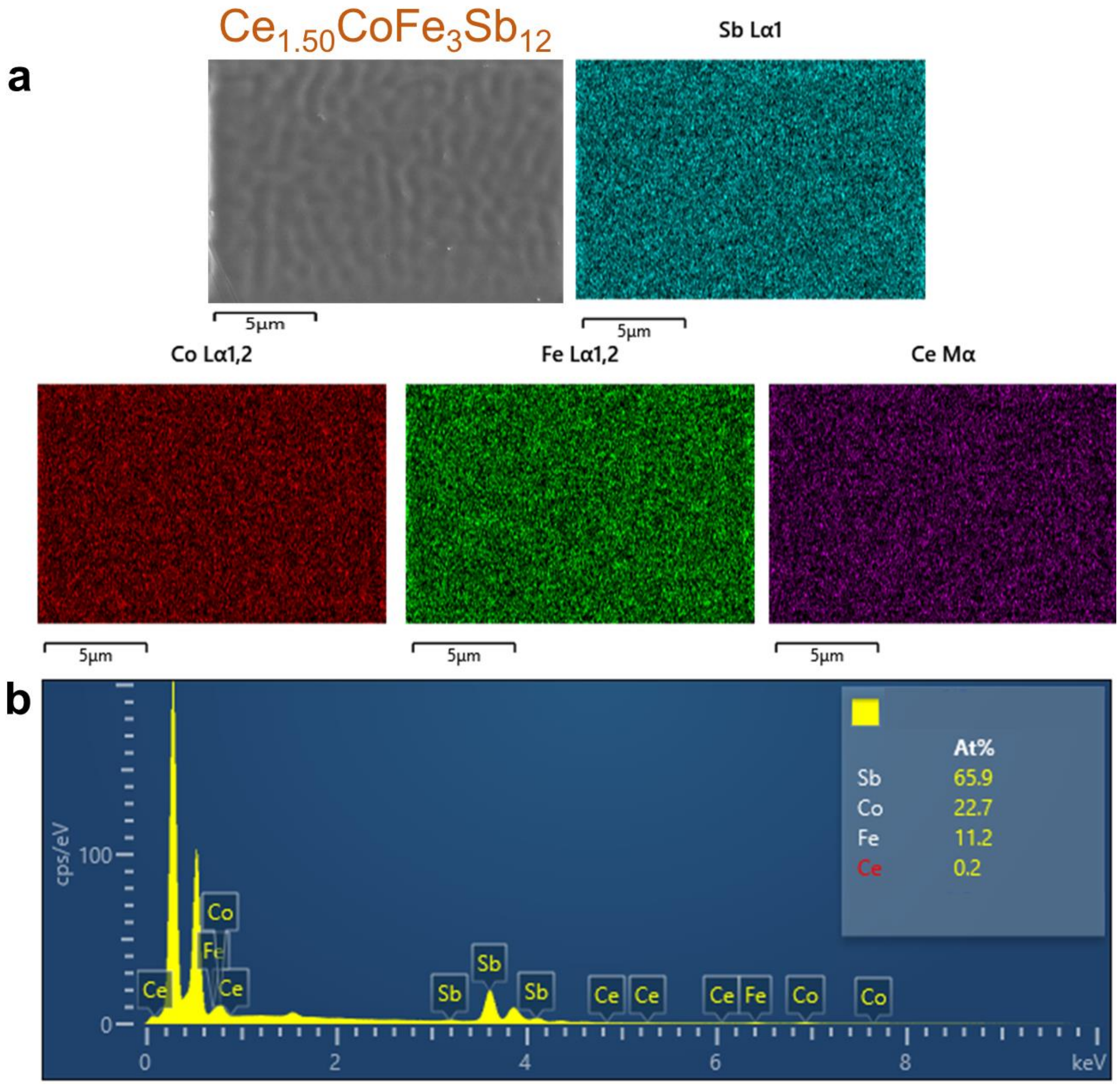

Supplementary Fig. 9 The surface morphology and composition of $Ce_{1.50}Fe_3CoSb_{12}$ skutterudite thin film. (**a**) SEM image of $Ce_{1.50}Fe_3CoSb_{12}$ skutterudite thin film from a top view and corresponding EDS maps for Sb, Co, Fe, and Ce. (**b**) Corresponding EDS composition results taken from the EDS maps.

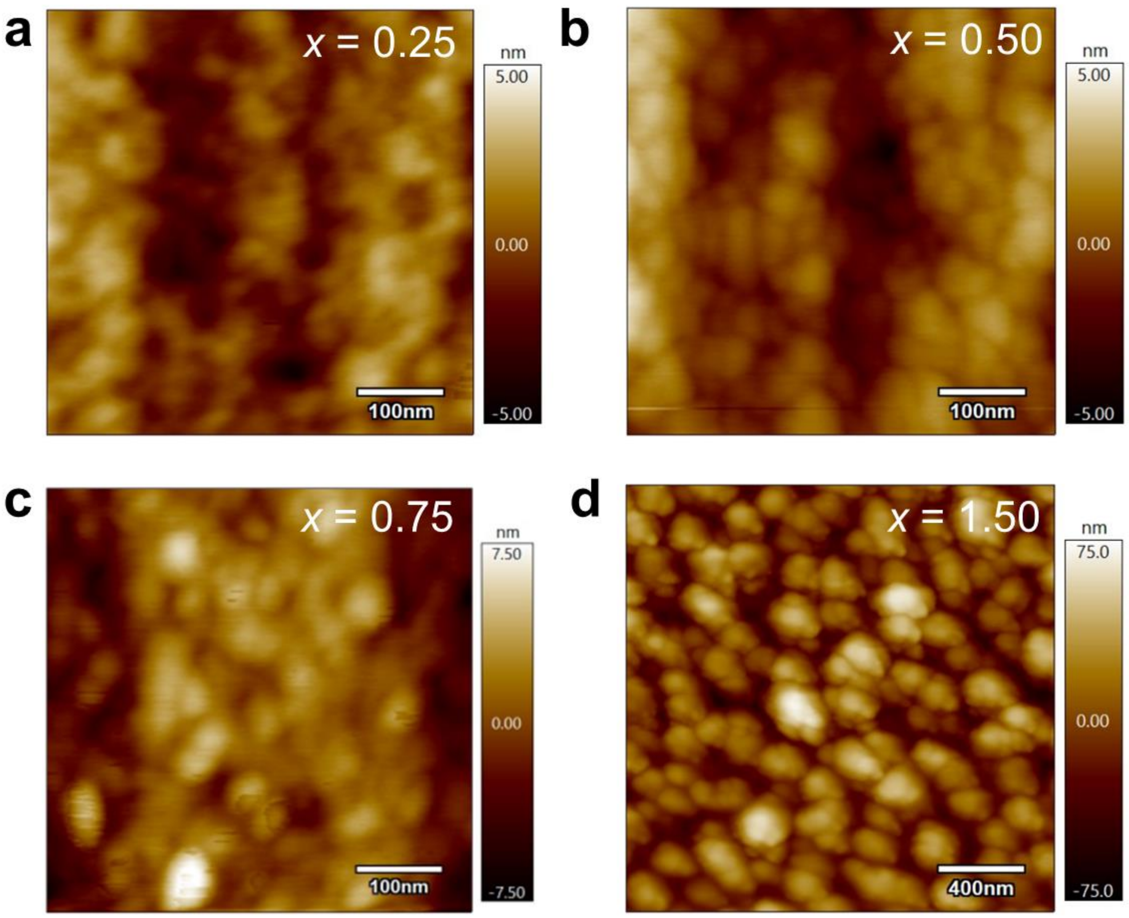

Supplementary Fig. 10 Atomic force microscopy (AFM) images of $Ce_xFe_3CoSb_{12}$ thin films. (**a**) x = 0.25, (**b**) x = 0.50, (**c**) x = 0.75, and (**d**) x = 1.50.

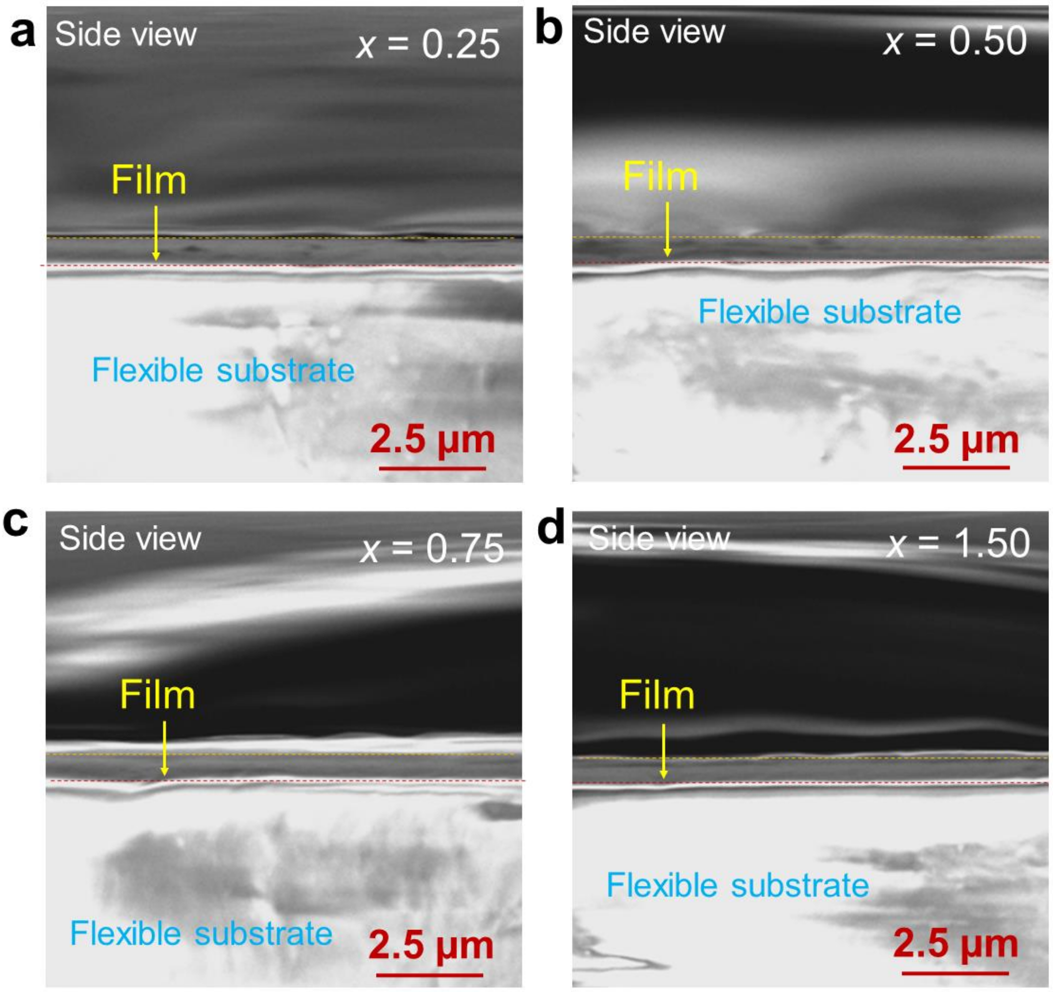

Supplementary Fig. 11 SEM images of $Ce_xFe_3CoSb_{12}$ thin films from cross-sectional views. (**a**) x = 0.25, (**b**) x = 0.50, (**c**) x = 0.75, and (**d**) x = 1.50.

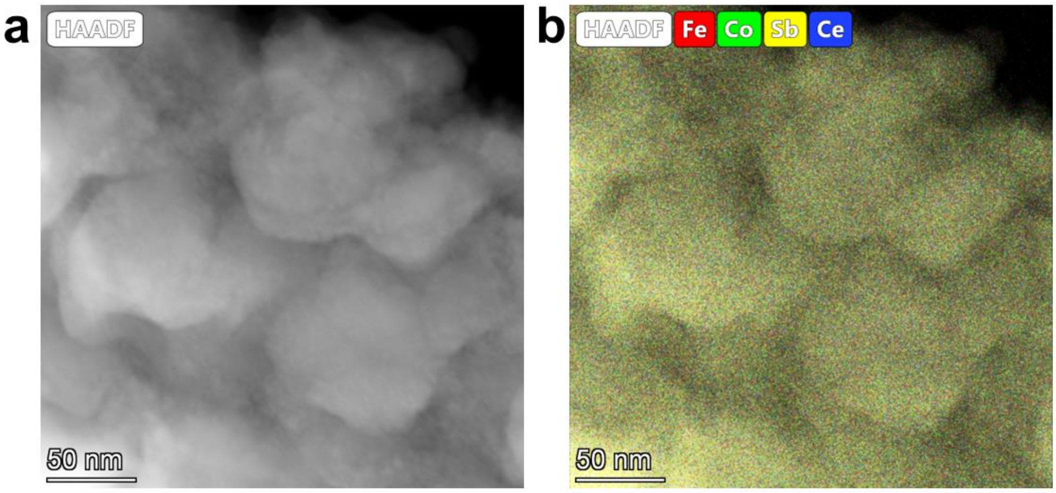

Supplementary Fig. 12 The microstructure and composition of $Ce_{1.25}Fe_3CoSb_{12}$ skutterudite thin film. (**a**) Transmission electron microscopy (TEM) high-angle annular dark-field (HAADF) image of the $Ce_{1.25}Fe_3CoSb_{12}$ thin film and (**b**) corresponding EDS map for overlapping elements.

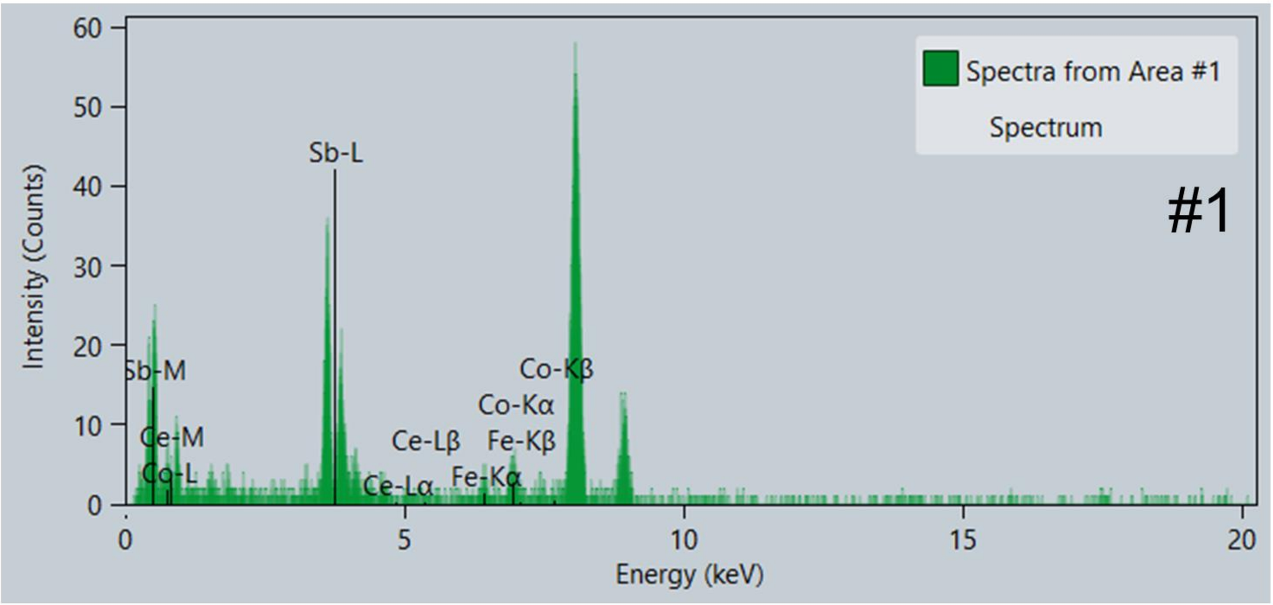

Element	Family	Atomic Fraction (%)	Atomic Error (%)	Mass Fraction (%)	Mass Error (%)	Fit error (%)
Fe	K	6.11	1.27	3.10	0.49	10.73
Co	K	12.25	2.73	6.55	1.17	6.58
Sb	L	80.65	16.01	89.09	13.09	0.86
Ce	L	0.99	0.40	1.26	0.48	35.67

Supplementary Fig. 13 Detailed EDS spot result for #1 taken from **Fig. 3a**.

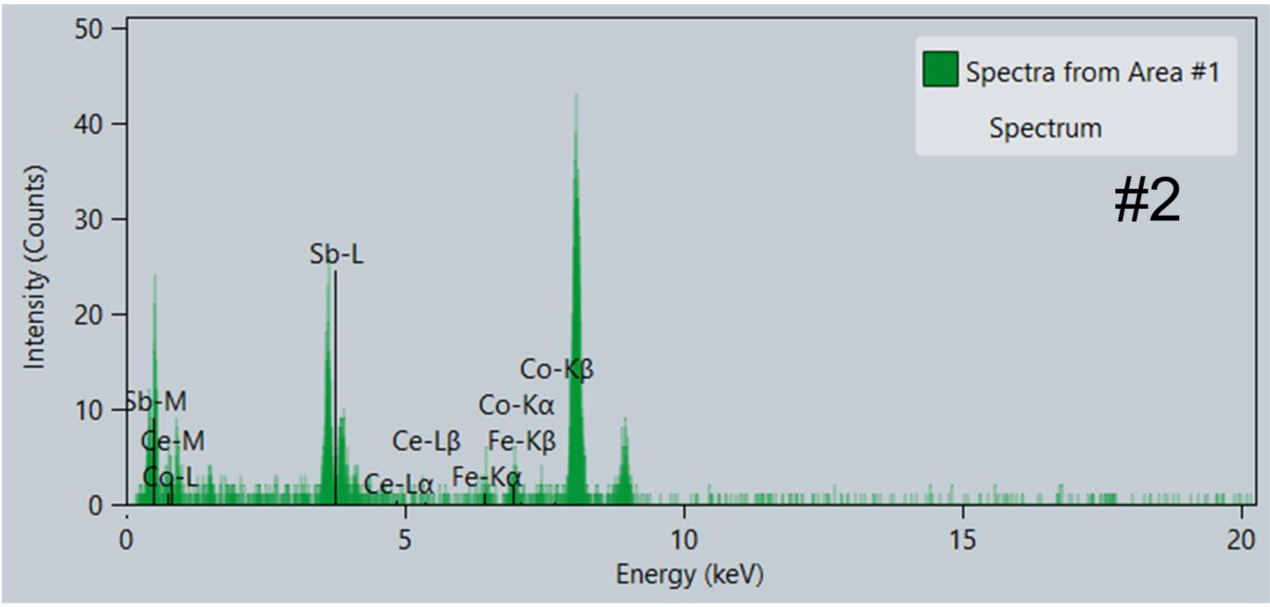

Element	Family	Atomic Fraction (%)	Atomic Error (%)	Mass Fraction (%)	Mass Error (%)	Fit error (%)
Fe	K	7.57	1.67	3.89	0.69	13.65
Co	K	13.43	3.04	7.28	1.35	8.51
Sb	L	77.02	15.05	86.27	12.57	1.23
Ce	L	1.98	0.69	2.56	0.82	29.34

Supplementary Fig. 14 Detailed EDS spot result for #2 taken from **Fig. 3a**.

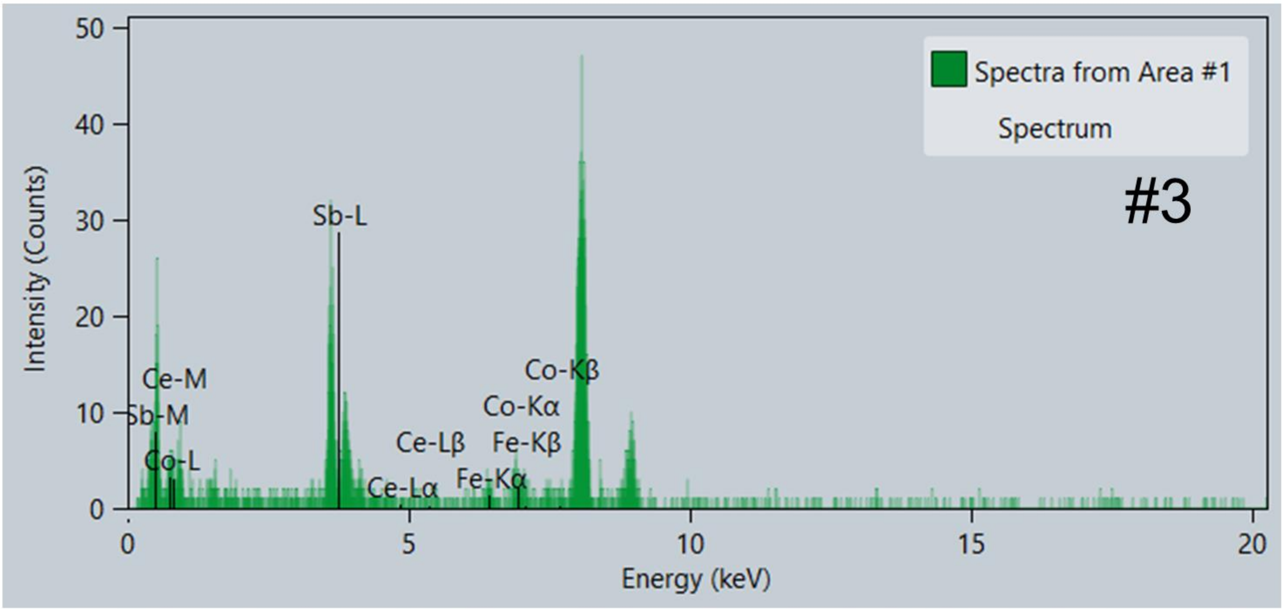

Element	Family	Atomic Fraction (%)	Atomic Error (%)	Mass Fraction (%)	Mass Error (%)	Fit error (%)
Fe	K	8.10	1.68	4.16	0.67	11.24
Co	K	12.58	2.84	6.81	1.25	8.18
Sb	L	77.79	15.24	87.06	12.71	1.12
Ce	L	1.53	0.57	1.97	0.69	32.59

Supplementary Fig. 15 Detailed EDS spot result for #3 taken from **Fig. 3a**.

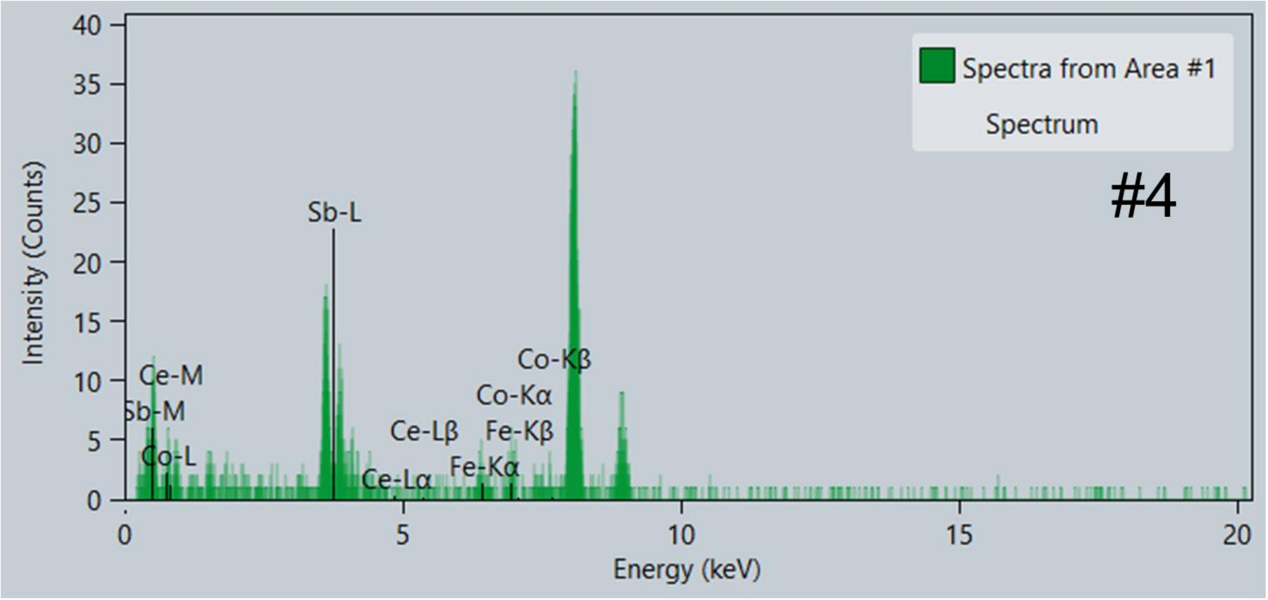

Element	Family	Atomic Fraction (%)	Atomic Error (%)	Mass Fraction (%)	Mass Error (%)	Fit error (%)
Fe	K	10.45	2.15	5.38	0.85	10.89
Co	K	10.64	2.54	5.78	1.15	11.36
Sb	L	77.15	15.10	86.57	12.64	1.36
Ce	L	1.76	0.71	2.28	0.86	35.66

Supplementary Fig. 16 Detailed EDS spot result for #4 taken from **Fig. 3a**.

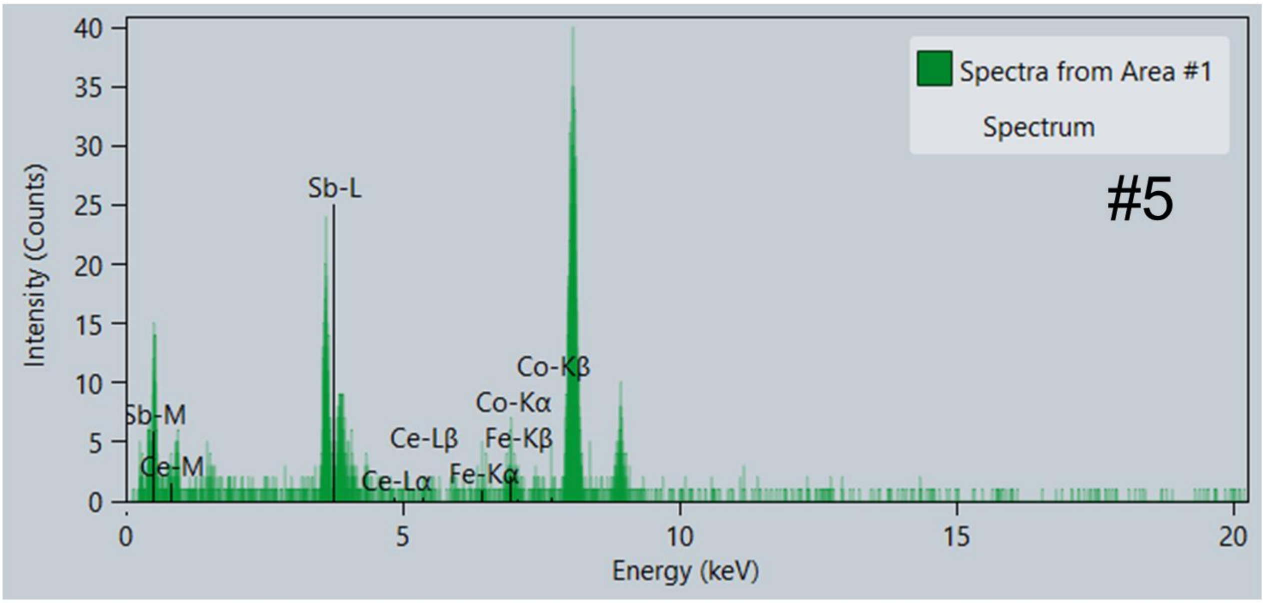

Element	Family	Atomic Fraction (%)	Atomic Error (%)	Mass Fraction (%)	Mass Error (%)	Fit error (%)
Fe	K	5.84	1.43	3.00	0.62	17.17
Co	K	15.27	3.40	8.28	1.50	7.55
Sb	L	77.03	15.08	86.32	12.58	1.22
Ce	L	1.86	0.67	2.40	0.80	30.57

Supplementary Fig. 17 Detailed EDS spot result for #5 taken from **Fig. 3a**.

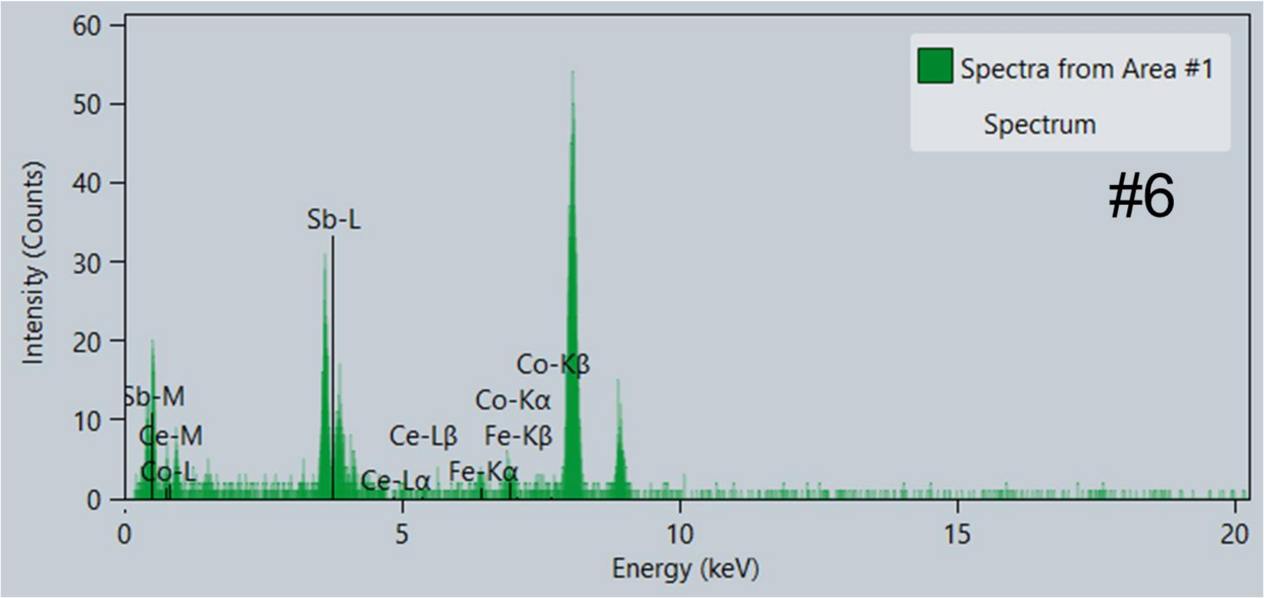

Element	Family	Atomic Fraction (%)	Atomic Error (%)	Mass Fraction (%)	Mass Error (%)	Fit error (%)
Fe	K	7.46	1.54	3.80	0.60	10.77
Co	K	11.64	2.63	6.25	1.14	7.76
Sb	L	79.86	15.81	88.63	13.01	1.04
Ce	L	1.03	0.48	1.32	0.59	42.91

Supplementary Fig. 18 Detailed EDS spot result for #6 taken from **Fig. 3a**.

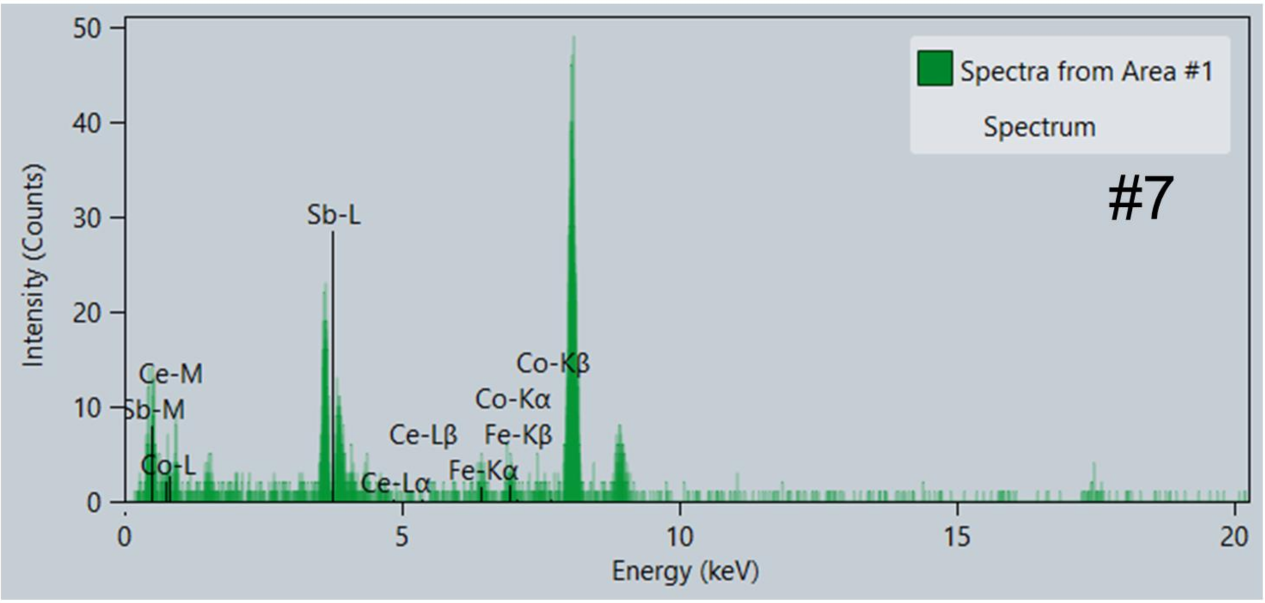

Element	Family	Atomic Fraction (%)	Atomic Error (%)	Mass Fraction (%)	Mass Error (%)	Fit error (%)
Fe	K	9.57	1.94	4.91	0.76	10.15
Co	K	10.77	2.48	5.83	1.10	9.27
Sb	L	78.50	15.43	87.77	12.84	1.01
Ce	L	1.16	0.55	1.49	0.68	43.97

Supplementary Fig. 19 Detailed EDS spot result for #7 taken from **Fig. 3a**.

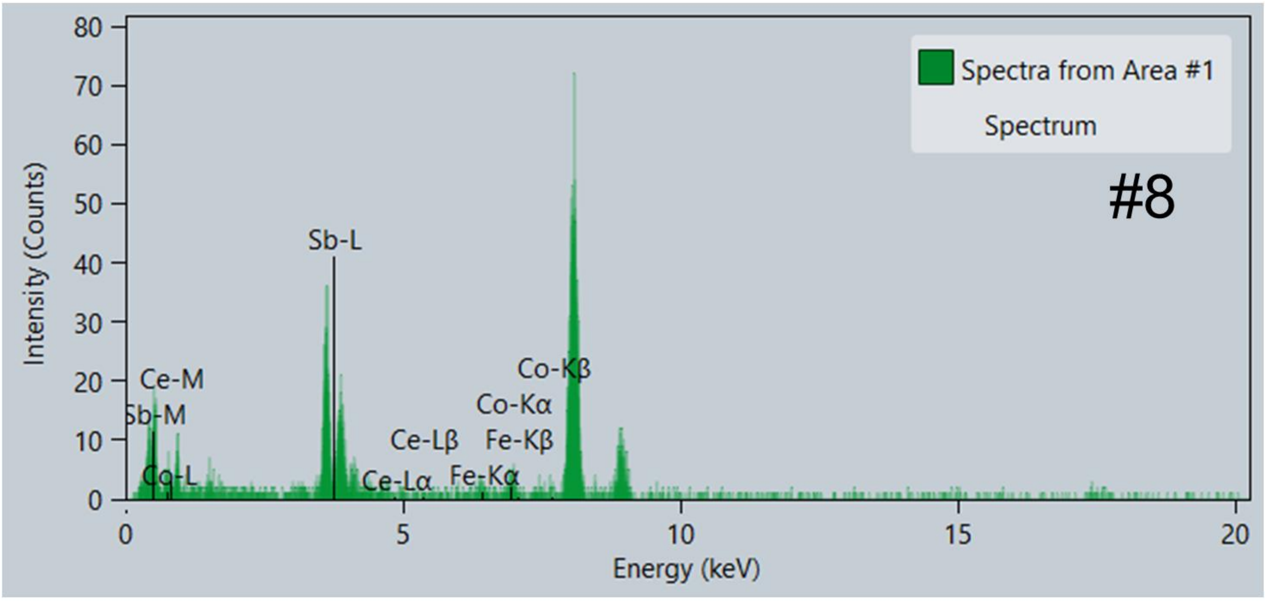

Element	Family	Atomic Fraction (%)	Atomic Error (%)	Mass Fraction (%)	Mass Error (%)	Fit error (%)
Fe	K	5.77	1.25	2.88	0.49	12.04
Co	K	10.03	2.29	5.29	0.97	7.72
Sb	L	83.05	16.68	90.39	13.37	0.98
Ce	L	1.15	0.44	1.44	0.51	32.78

Supplementary Fig. 20 Detailed EDS spot result for #8 taken from **Fig. 3a**.

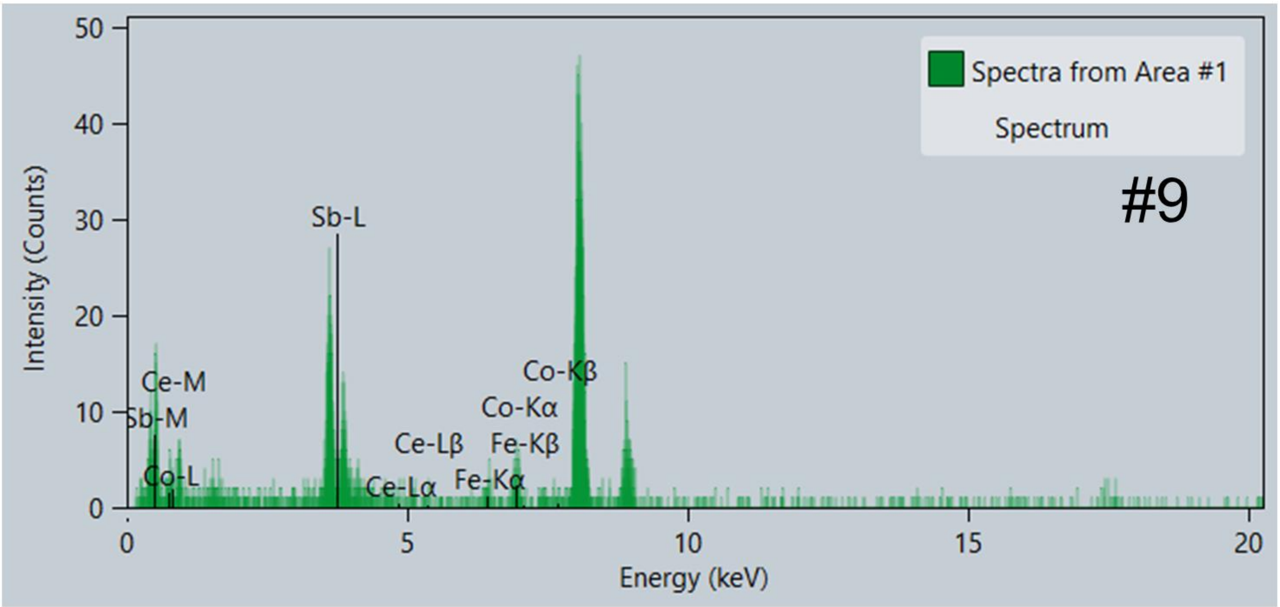

Element	Family	Atomic Fraction (%)	Atomic Error (%)	Mass Fraction (%)	Mass Error (%)	Fit error (%)
Fe	K	6.94	1.49	3.57	0.61	12.67
Co	K	14.37	3.19	7.81	1.40	7.34
Sb	L	76.82	14.98	86.21	12.55	1.19
Ce	L	1.87	0.61	2.41	0.73	27.06

Supplementary Fig. 21 Detailed EDS spot result for #9 taken from **Fig. 3a**.

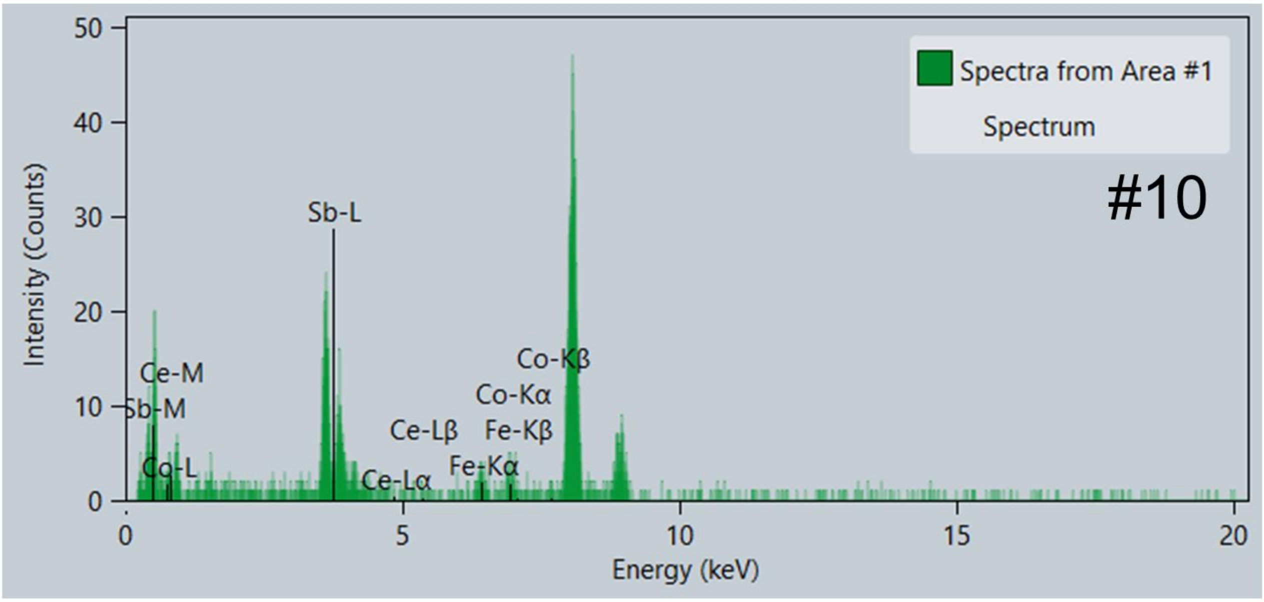

Element	Family	Atomic Fraction (%)	Atomic Error (%)	Mass Fraction (%)	Mass Error (%)	Fit error (%)
Fe	K	10.84	2.11	5.59	0.81	8.80
Co	K	10.59	2.44	5.76	1.10	9.71
Sb	L	77.16	15.05	86.82	12.64	1.06
Ce	L	1.41	0.56	1.83	0.69	35.75

Supplementary Fig. 22 Detailed EDS spot result for #10 taken from **Fig. 3a**.

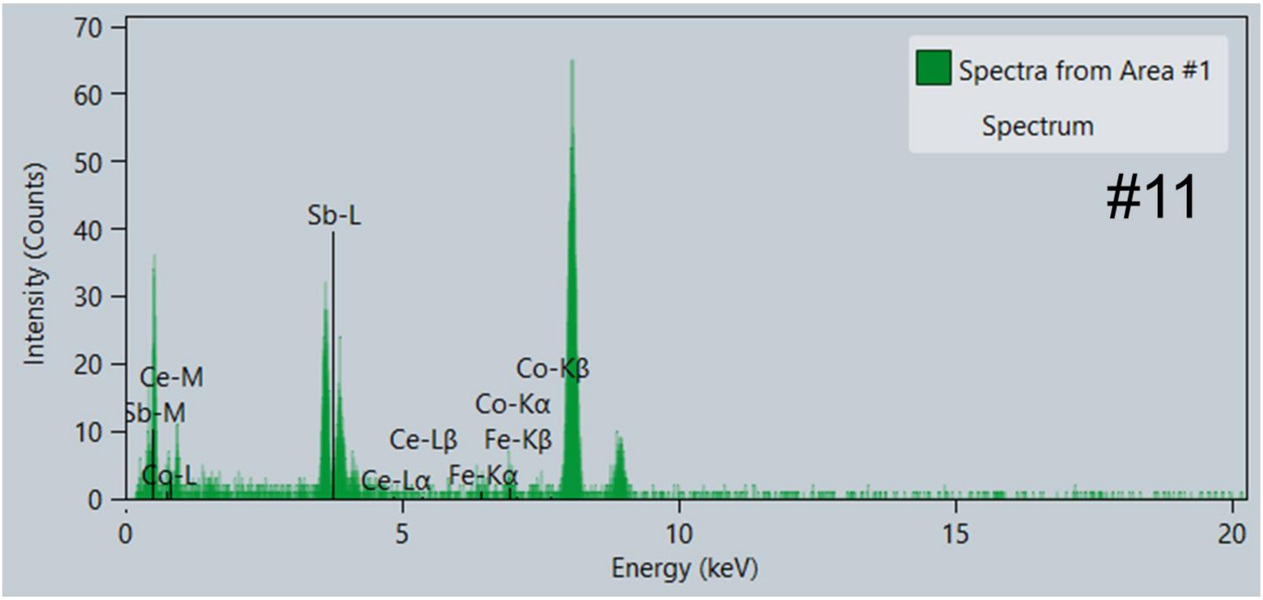

Element	Family	Atomic Fraction (%)	Atomic Error (%)	Mass Fraction (%)	Mass Error (%)	Fit error (%)
Fe	K	5.24	1.19	2.58	0.47	13.59
Co	K	8.11	1.92	4.21	0.81	9.65
Sb	L	85.36	17.33	91.61	13.62	0.88
Ce	L	1.29	0.47	1.59	0.54	30.86

Supplementary Fig. 23 Detailed EDS spot result for #11 taken from **Fig. 3a**.

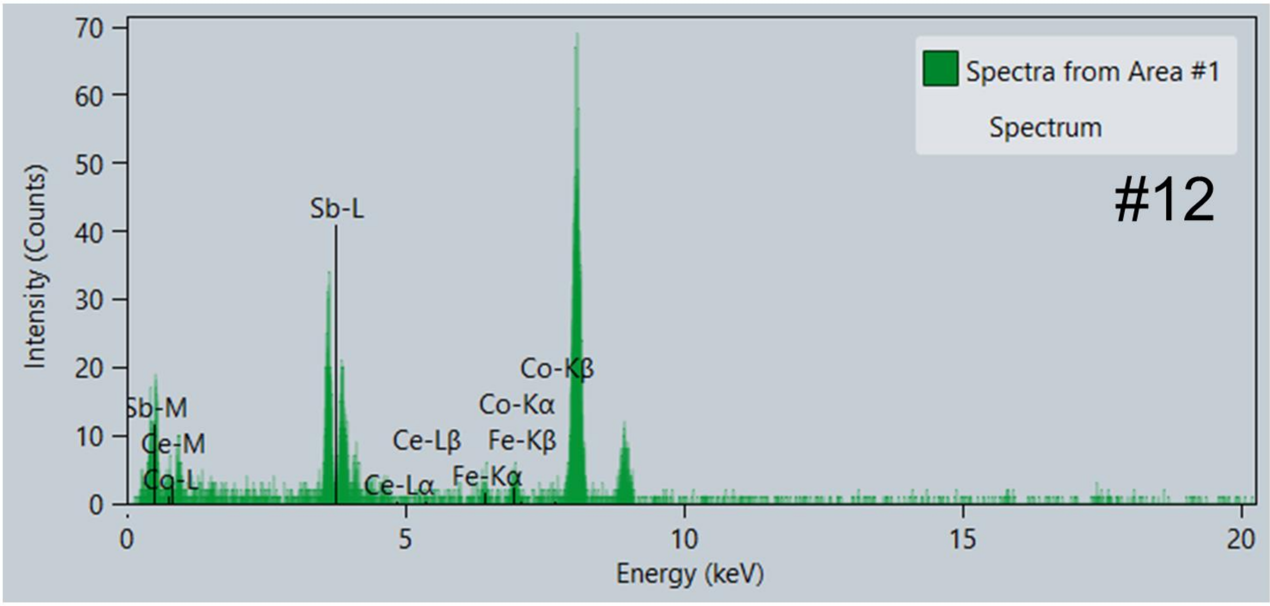

Element	Family	Atomic Fraction (%)	Atomic Error (%)	Mass Fraction (%)	Mass Error (%)	Fit error (%)
Fe	K	6.71	1.38	3.38	0.52	10.12
Co	K	10.54	2.37	5.60	1.01	7.08
Sb	L	81.76	16.33	89.77	13.24	1.00
Ce	L	0.98	0.41	1.24	0.49	37.69

Supplementary Fig. 24 Detailed EDS spot result for #12 taken from **Fig. 3a**.

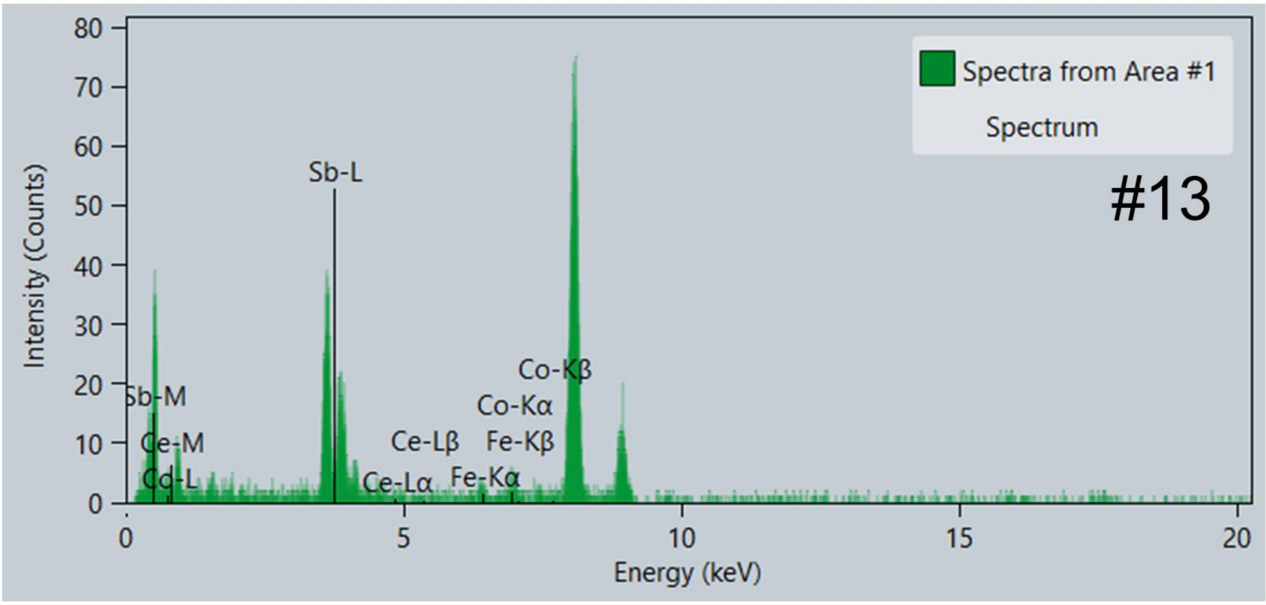

Element	Family	Atomic Fraction (%)	Atomic Error (%)	Mass Fraction (%)	Mass Error (%)	Fit error (%)
Fe	K	5.64	1.18	2.76	0.43	10.10
Co	K	6.81	1.62	3.52	0.68	9.63
Sb	L	86.10	17.50	91.94	13.67	0.81
Ce	L	1.44	0.42	1.77	0.45	21.09

Supplementary Fig. 25 Detailed EDS spot result for #13 taken from **Fig. 3a**.

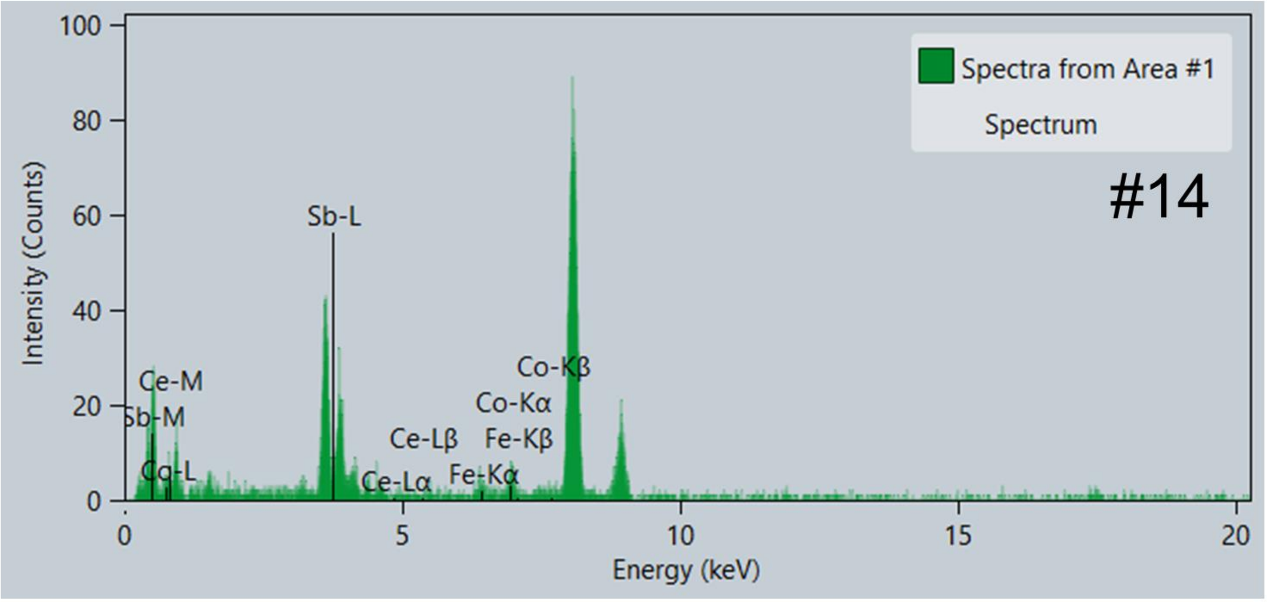

Element	Family	Atomic Fraction (%)	Atomic Error (%)	Mass Fraction (%)	Mass Error (%)	Fit error (%)
Fe	K	6.36	1.27	3.18	0.47	8.77
Co	K	9.64	2.16	5.09	0.91	6.34
Sb	L	83.11	16.68	90.62	13.40	0.82
Ce	L	0.88	0.32	1.11	0.37	30.95

Supplementary Fig. 26 Detailed EDS spot result for #14 taken from **Fig. 3a**.

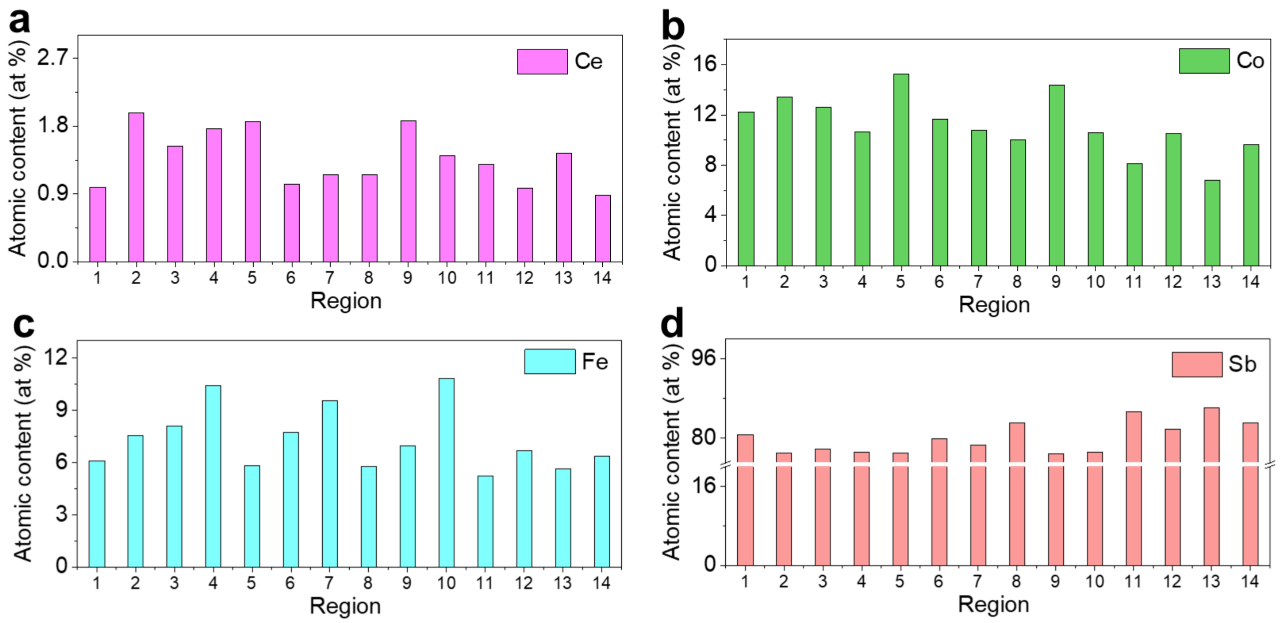

Supplementary Fig. 27 Determined atomic contents of the thin film. Atomic contents of (**a**) Ce, (**b**) Co, (**c**) Fe, and (**d**) Sb obtained within different EDS spot regions in **Fig. 3a**.

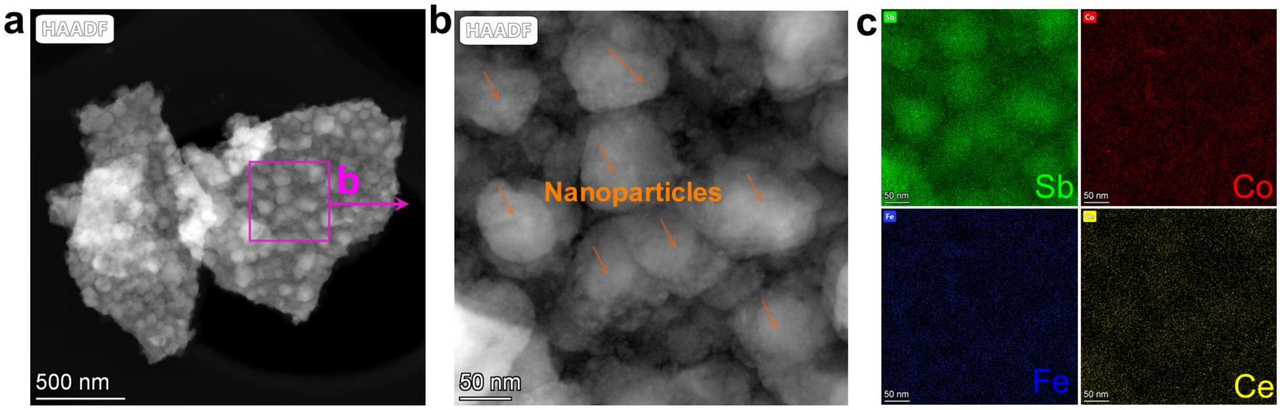

Supplementary Fig. 28 The microstructure and composition of $Ce_{1.25}Fe_3CoSb_{12}$ skutterudite thin film. (**a**) Low-magnification TEM image of a fragment taken from the $Ce_{1.25}Fe_3CoSb_{12}$ flexible thin film. (**b**) TEM image magnified from **a** and (**c**) corresponding EDS maps for Sb, Co, Fe, and Ce.

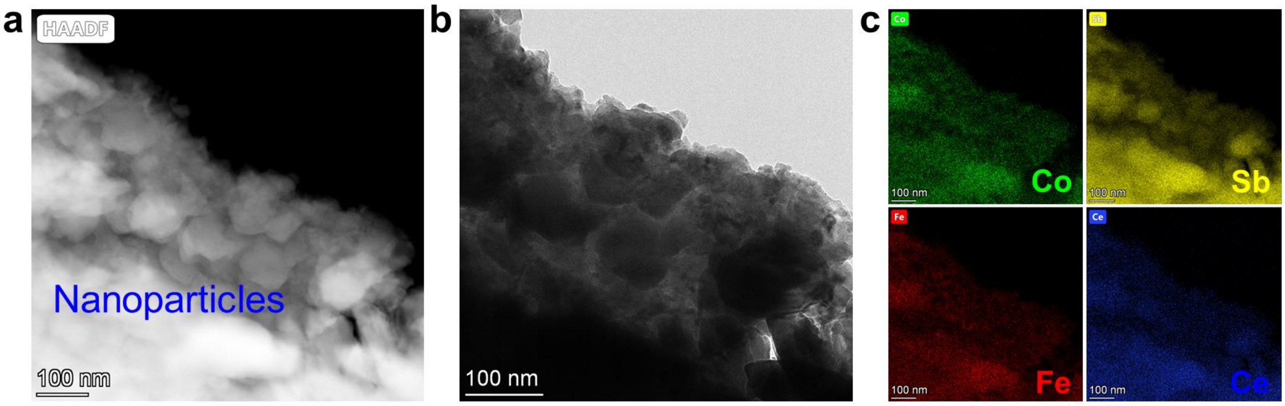

Supplementary Fig. 29 The microstructure and composition of $Ce_{1.25}Fe_3CoSb_{12}$ skutterudite thin film. (**a**) HAADF and (**b**) bright-field (BF) TEM images of a fragment taken from the $Ce_{1.25}Fe_3CoSb_{12}$ flexible thin film. (**c**) Corresponding EDS maps for Co, Sb, Fe, and Ce.

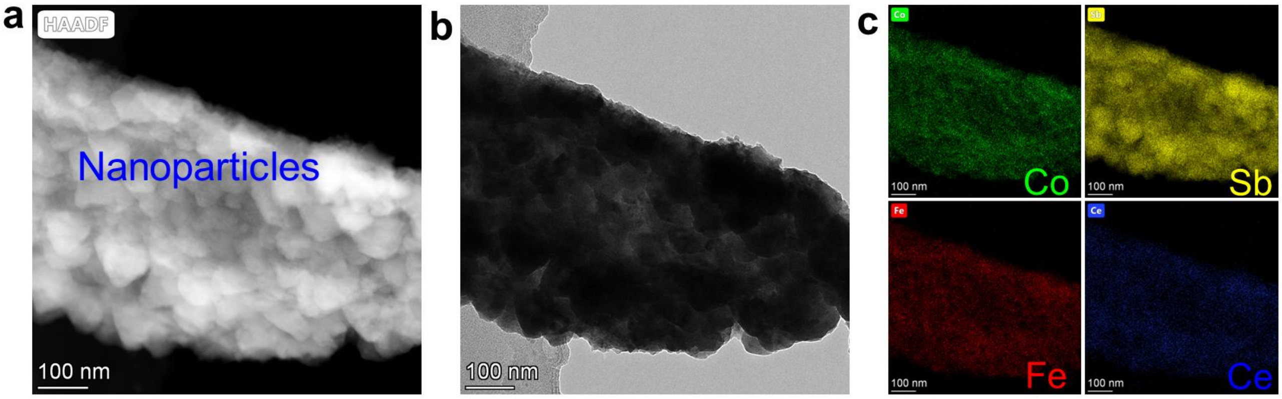

Supplementary Fig. 30 The microstructure and composition of $Ce_{1.25}Fe_3CoSb_{12}$ skutterudite thin film. (**a**) HAADF and (**b**) BF TEM images of a fragment taken from the $Ce_{1.25}Fe_3CoSb_{12}$ flexible thin film. (**c**) Corresponding EDS maps for Co, Sb, Fe, and Ce.

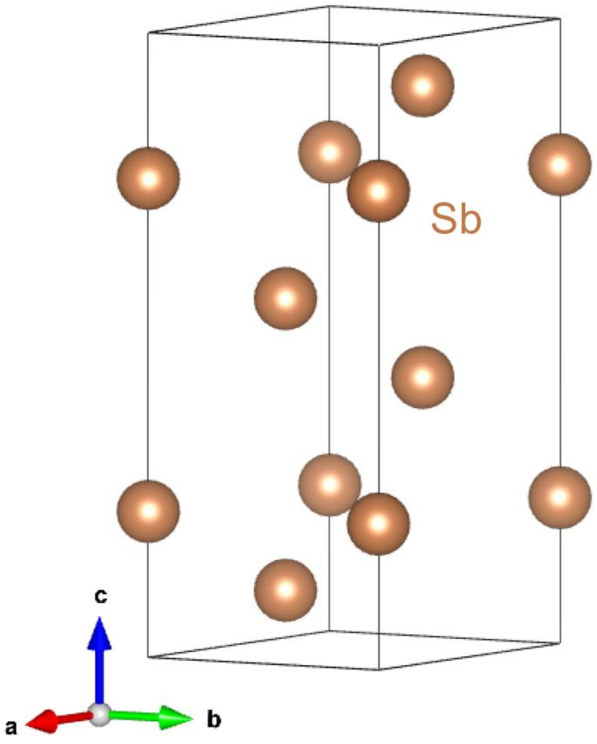

Supplementary Fig. 31 Unit cell of Sb element.

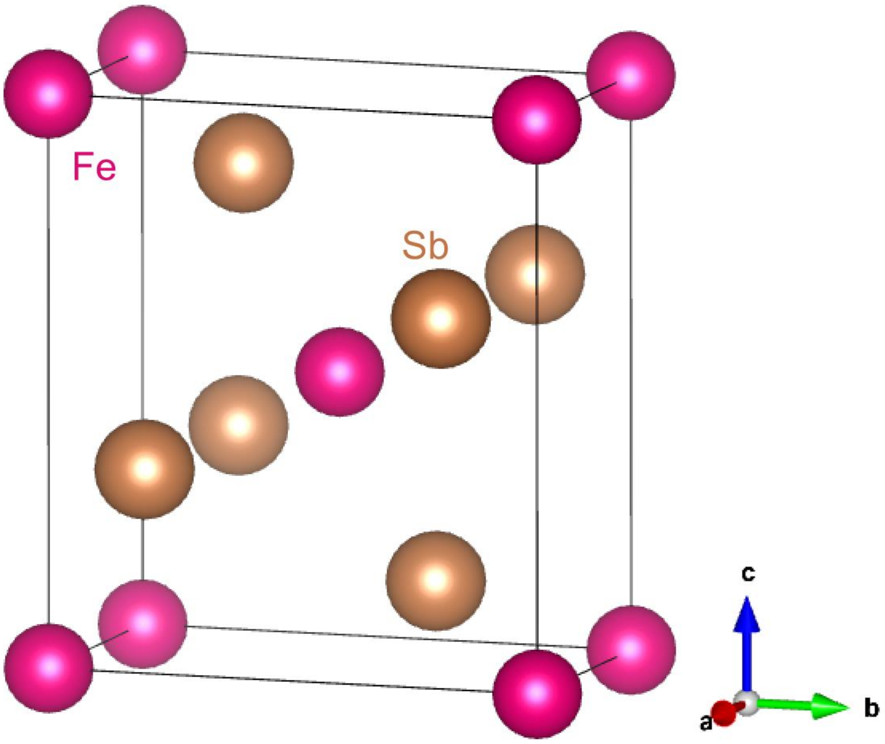

Supplementary Fig. 32 Unit cell of $FeSb_2$.

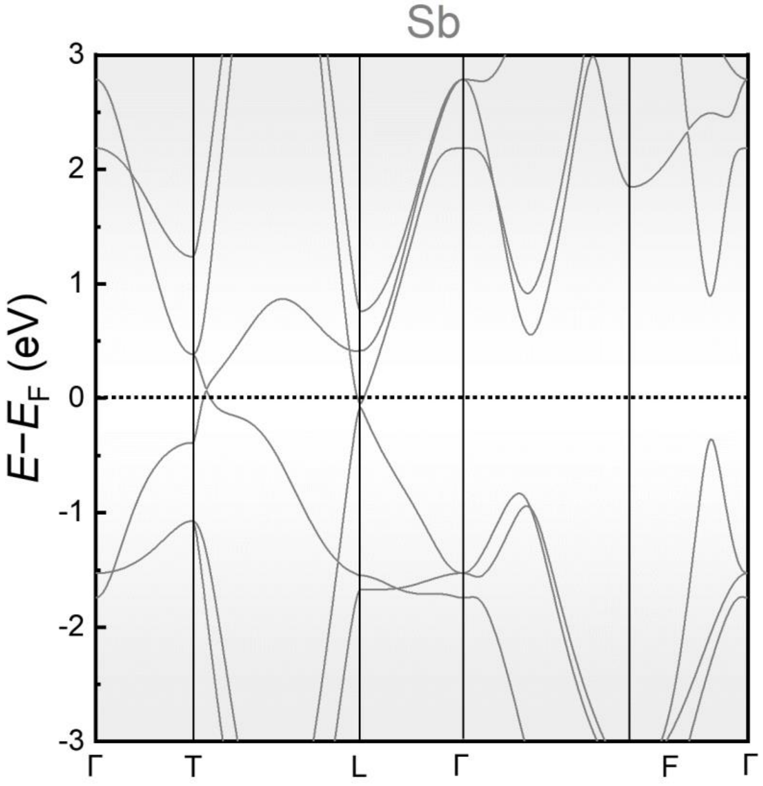

Supplementary Fig. 33 Calculated band structure of Sb element.

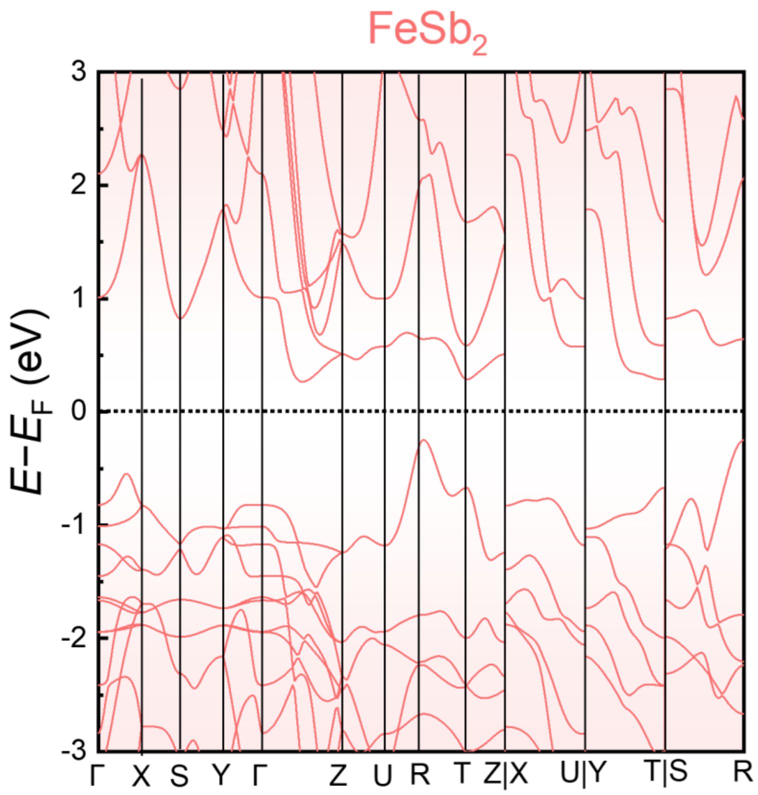

Supplementary Fig. 34 Calculated band structure of $FeSb_2$.

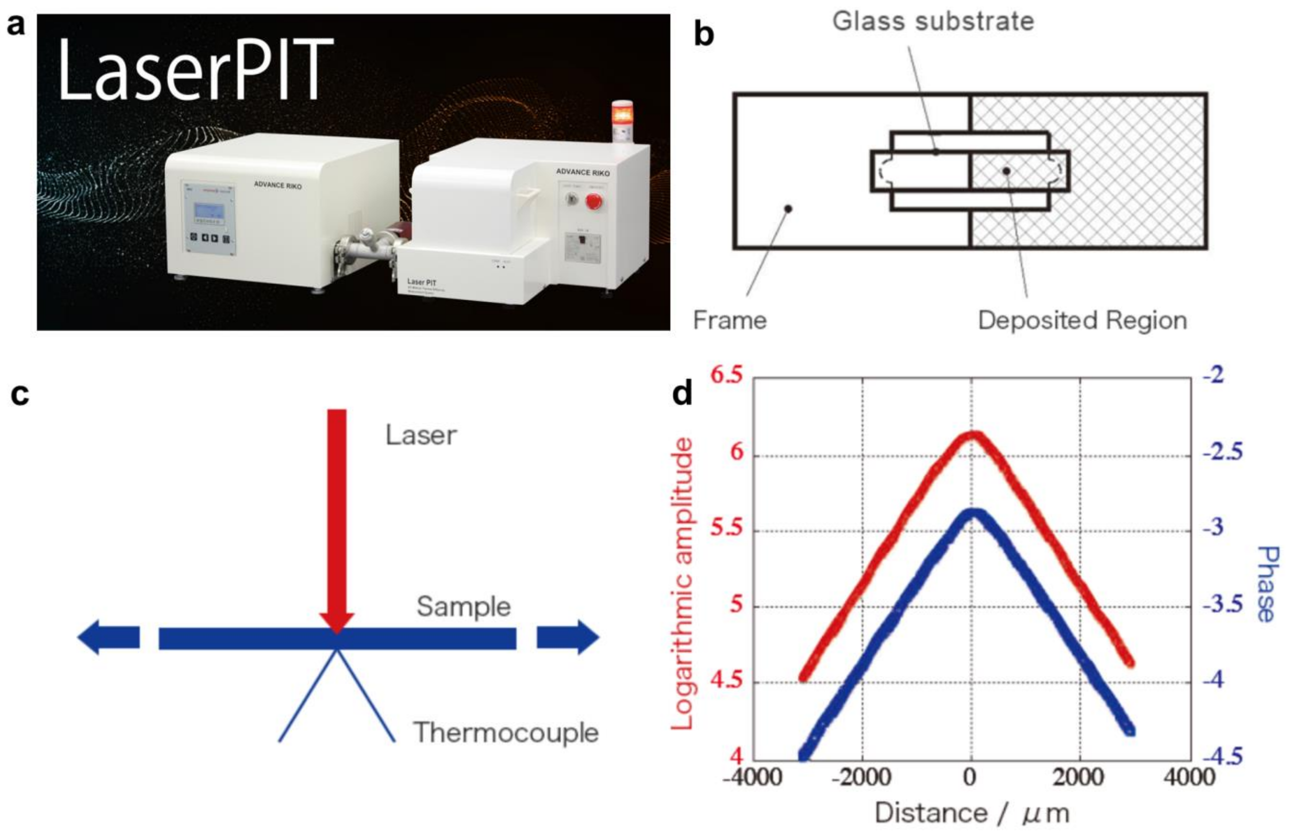

Supplementary Fig. 35 The measurement method (differential method) of thin film thermal conductivity. (**a**) Overview of Laser-PIT. (**b**) Schematic diagram of the thin film to be tested placed on the sample stage. (**c-d**) Testing principle. LaserPIT employs a glass substrate to measure the thermal conductivity of thin films. The method involves depositing the film on only half of one side of the glass substrate and measuring the coated and uncoated regions of the glass substrate. The thermal conductivity of the thin film is evaluated based on the measurement results from both regions, the thickness and volumetric specific heat capacity of the glass substrate, and the thickness and volumetric specific heat capacity of the thin film.

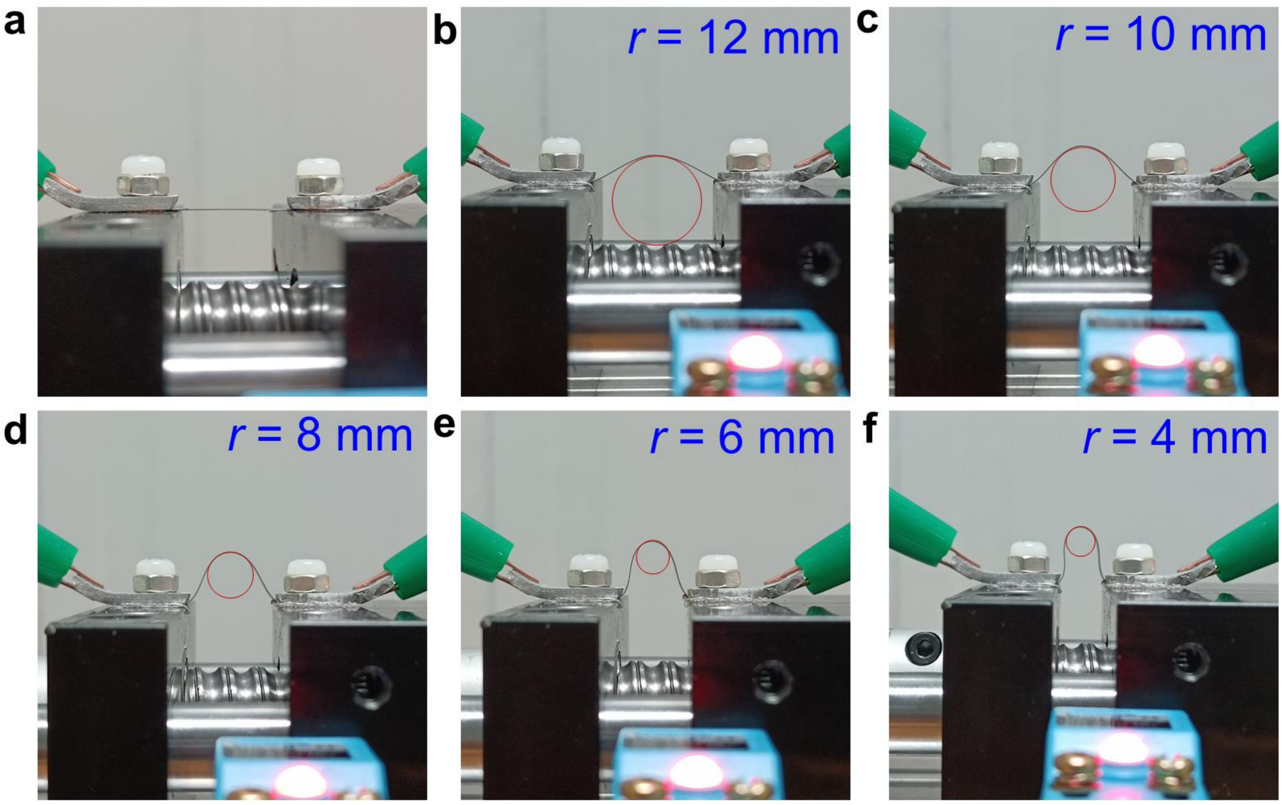

Supplementary Fig. 36 Photos that show the bending test. Photographs of flexible p-type $Ce_{1.25}Fe_3CoSb_{12}$ thin film during bending as a function of *r* for (**a**) not bending, (**b**) *r* = 12 mm, (**c**) *r* = 10 mm, (**d**) *r* = 8 mm, (**e**) *r* = 6 mm, and (**f**) *r* = 4 mm.

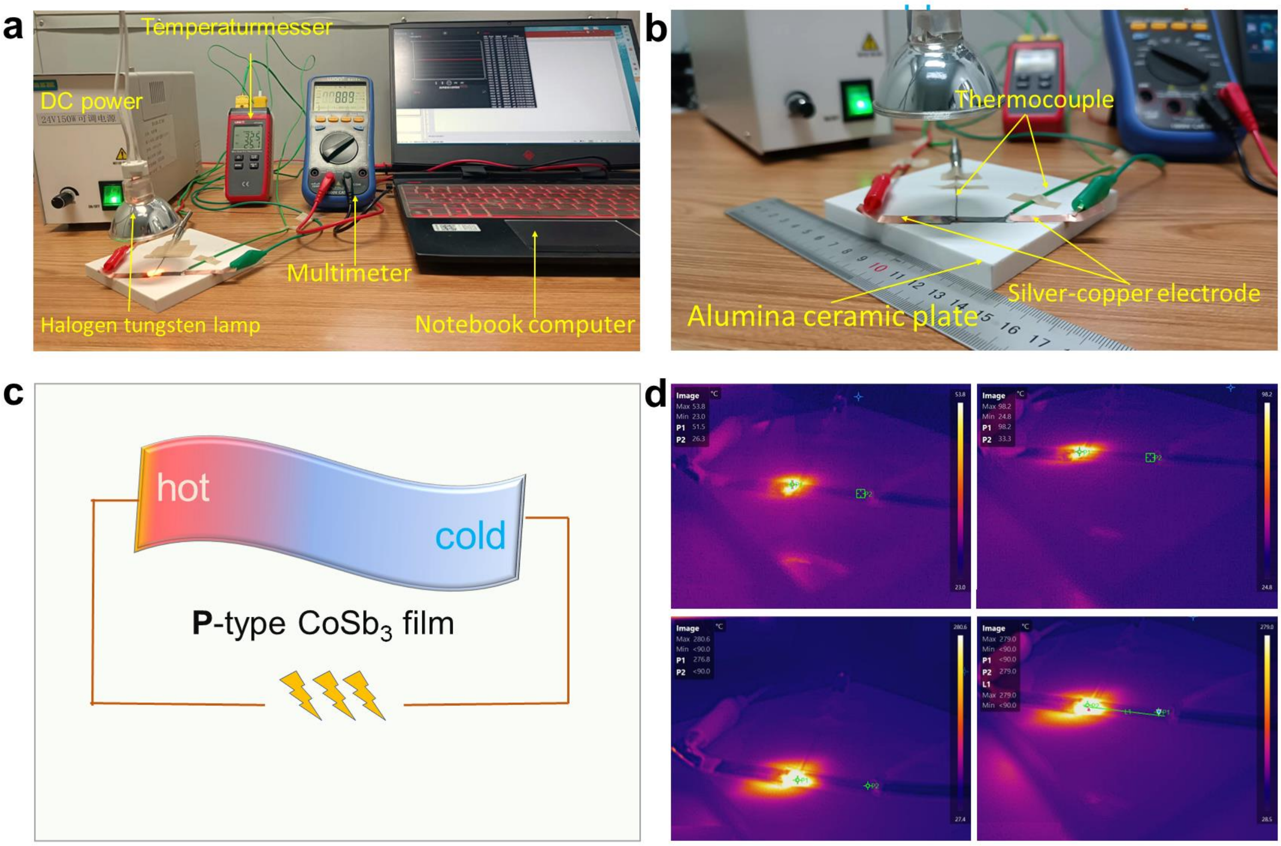

Supplementary Fig. 37 A photothermal-electric detection platform for thermoelectric thin film. (**a**) Photograph of light-induced thermal-electric signal detection platform. (**b**) Photograph of main components of the platform. (**c**) Principle of temperature detection of flexible thin films. (**d**) Infrared photos of temperature distribution of flexible film during light-induced thermal-electric detection at different temperatures.

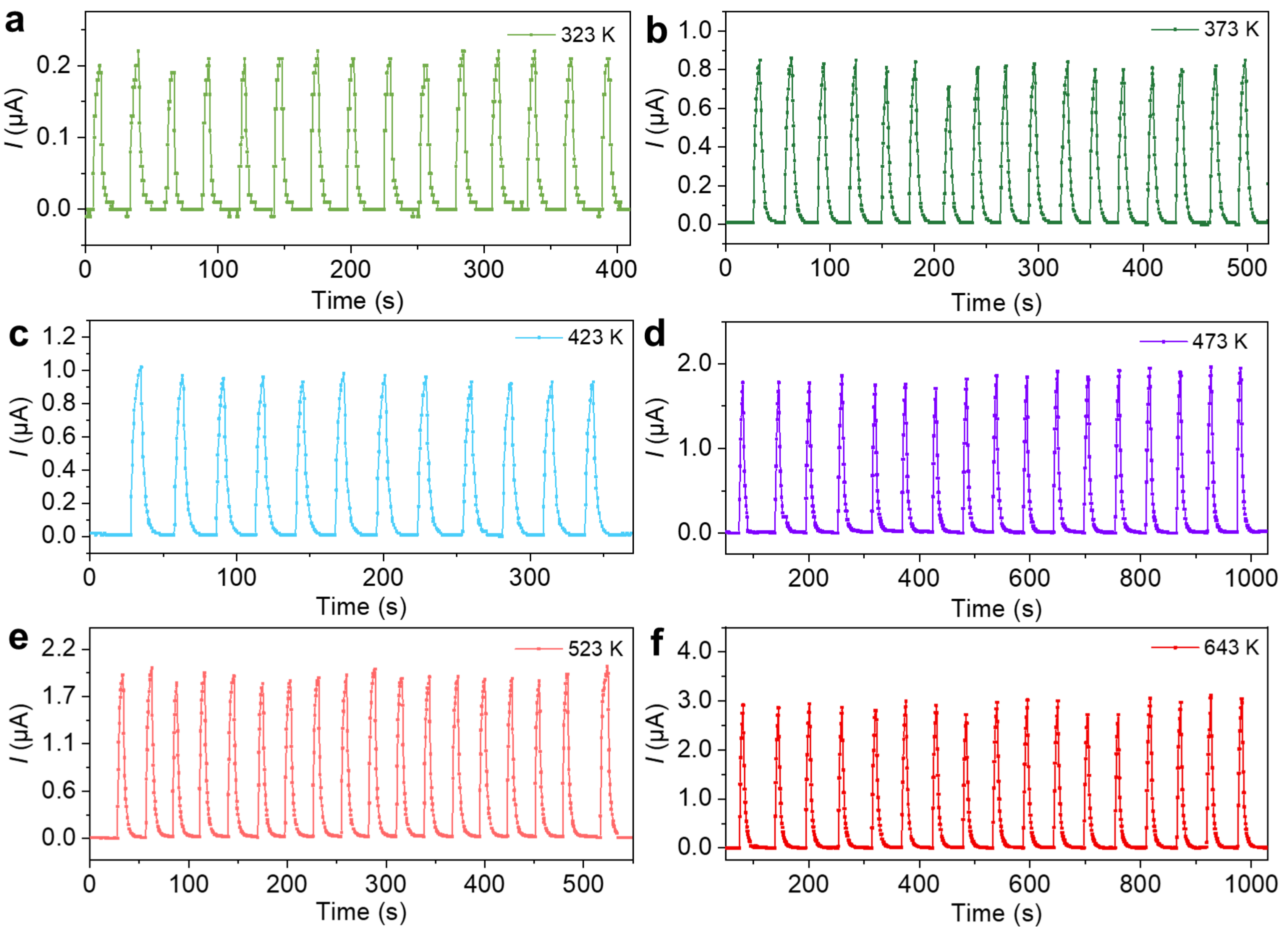

Supplementary Fig. 38 Measured current I varies over time when low-frequency light pulse modulation is applied at various light temperatures. (**a**) 323 K, (**b**) 373 K, (**c**) 423 K, (**d**) 473 K, (**e**) 523 K, and (**f**) 643 K.

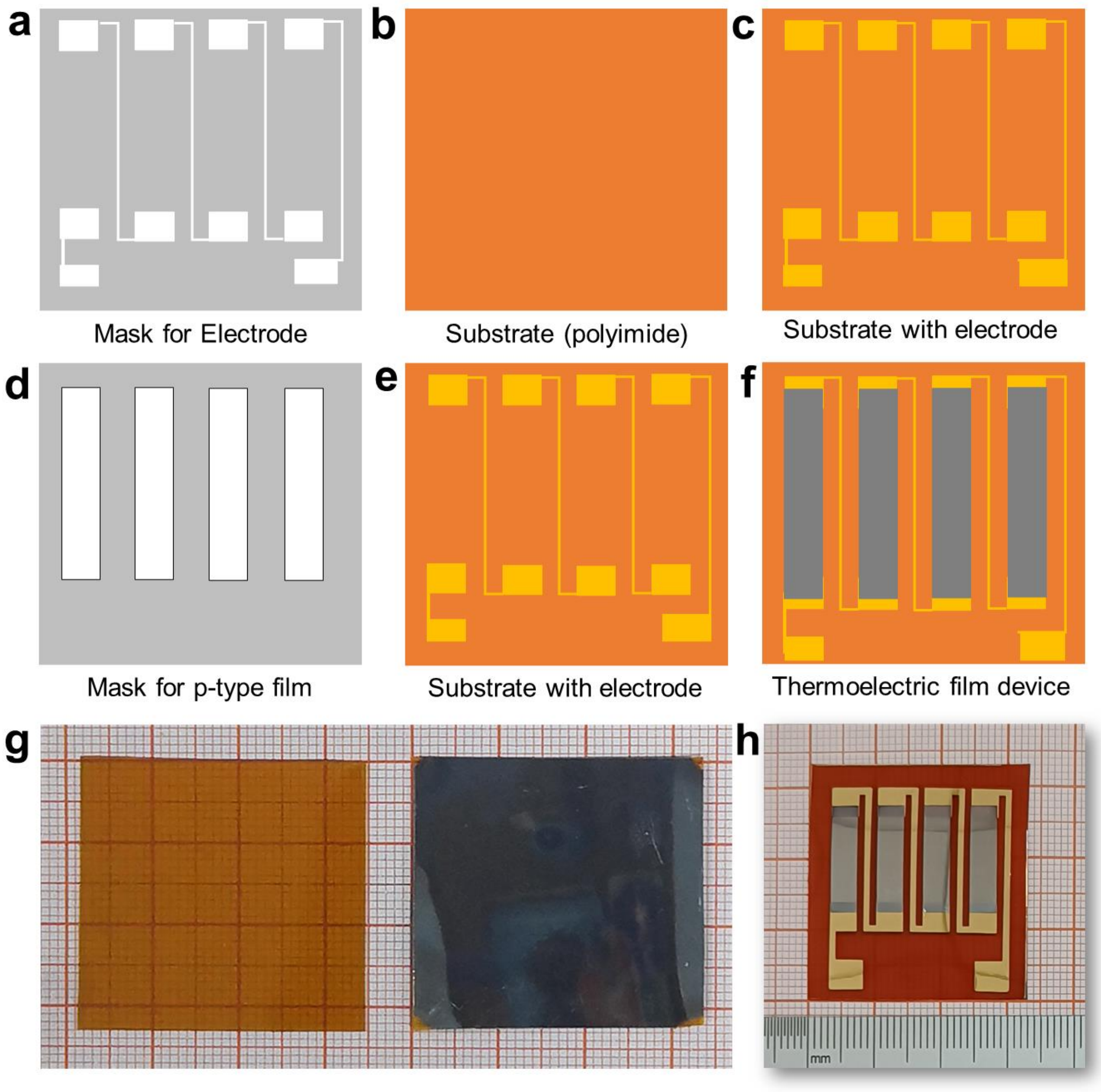

Supplementary Fig. 39 The main steps in the preparation process of flexible $Ce_{1.25}Fe_3CoSb_{12}$ thin-film-based thermoelectric devices. (**a**) Schematic diagram of electrode mask plate. (**b**) Schematic diagram of the flexible PI substrate. (**c**) Schematic diagram of gold electrode deposition on the substrate. (**d**) Schematic diagram of mask plate for thin films. (**e**) Substrate with gold electrodes for thin film deposition. (**f**) Schematic diagram of the complete device. (**g**) Comparison between blank substrate and substrate after thin film deposition. (**h**) Photograph of as-fabricated flexible thin-film-based device.

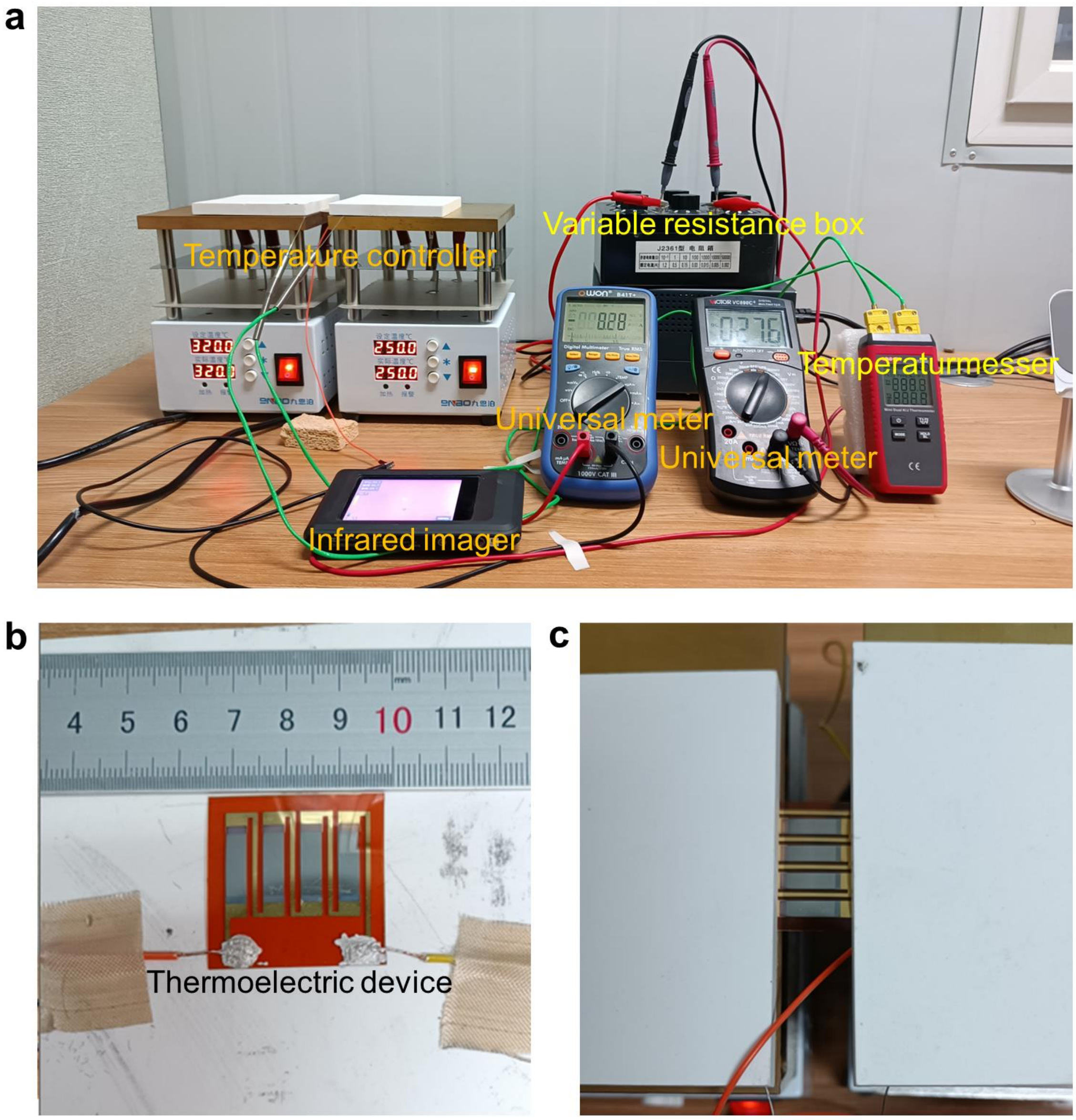

Supplementary Fig. 40 Photos of test platform for thin-film-based thermoelectric device. (**a**) Performance testing platform for flexible thin-film-based thermoelectric device. (**b**) External circuit connection for the device. (**c**) The setting of hot and cold sides during the device testing process.

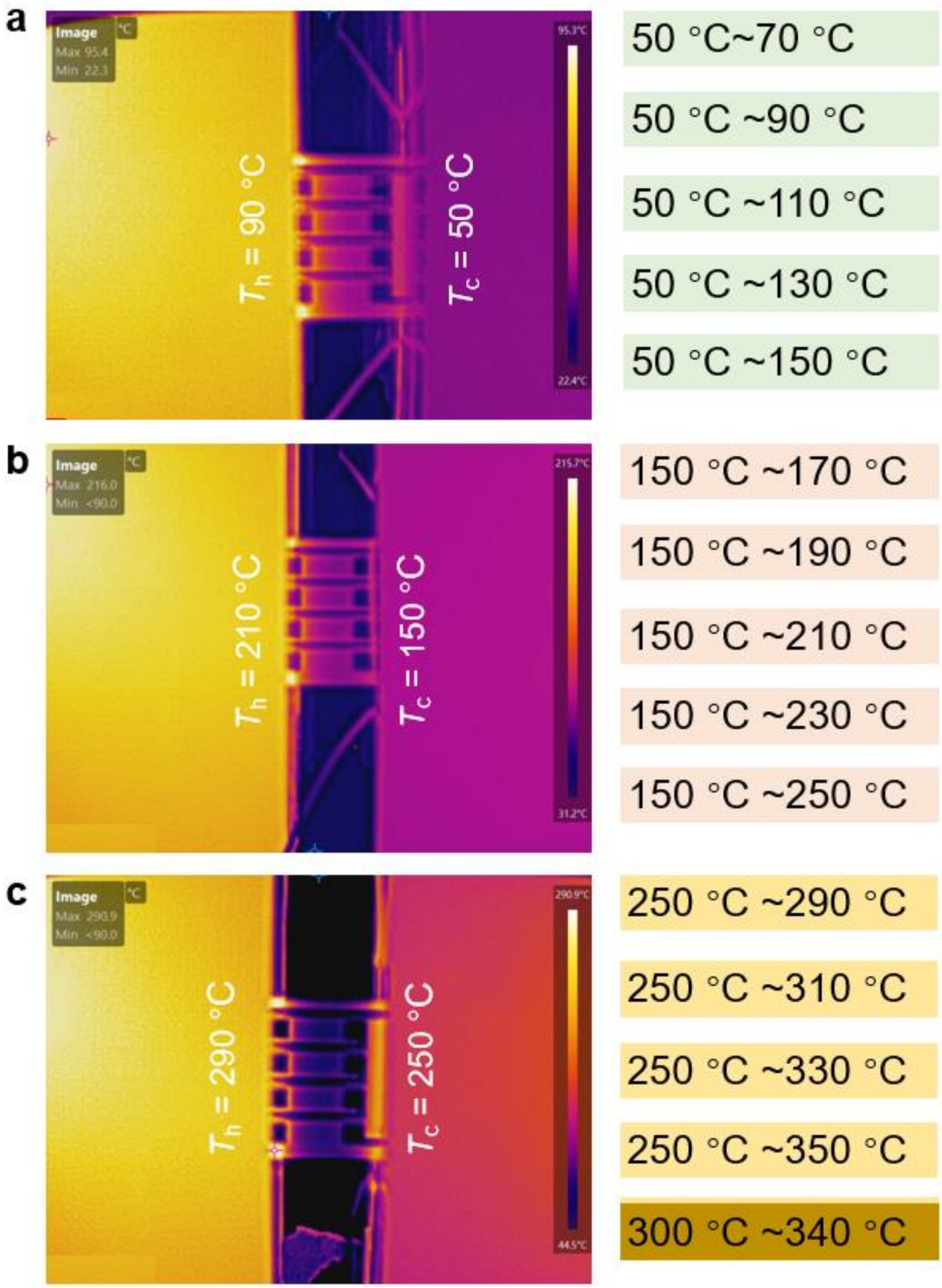

Supplementary Fig. 41 The infrared photos of the temperature distributions at the hot and cold sides of flexible thin-film-based devices during the performance testing process, along with their

corresponding temperature differentials as follows. (**a**) cold side kept at 50 °C, (**b**) cold side kept at 150 °C, and (**c**) cold side kept at 250 °C.

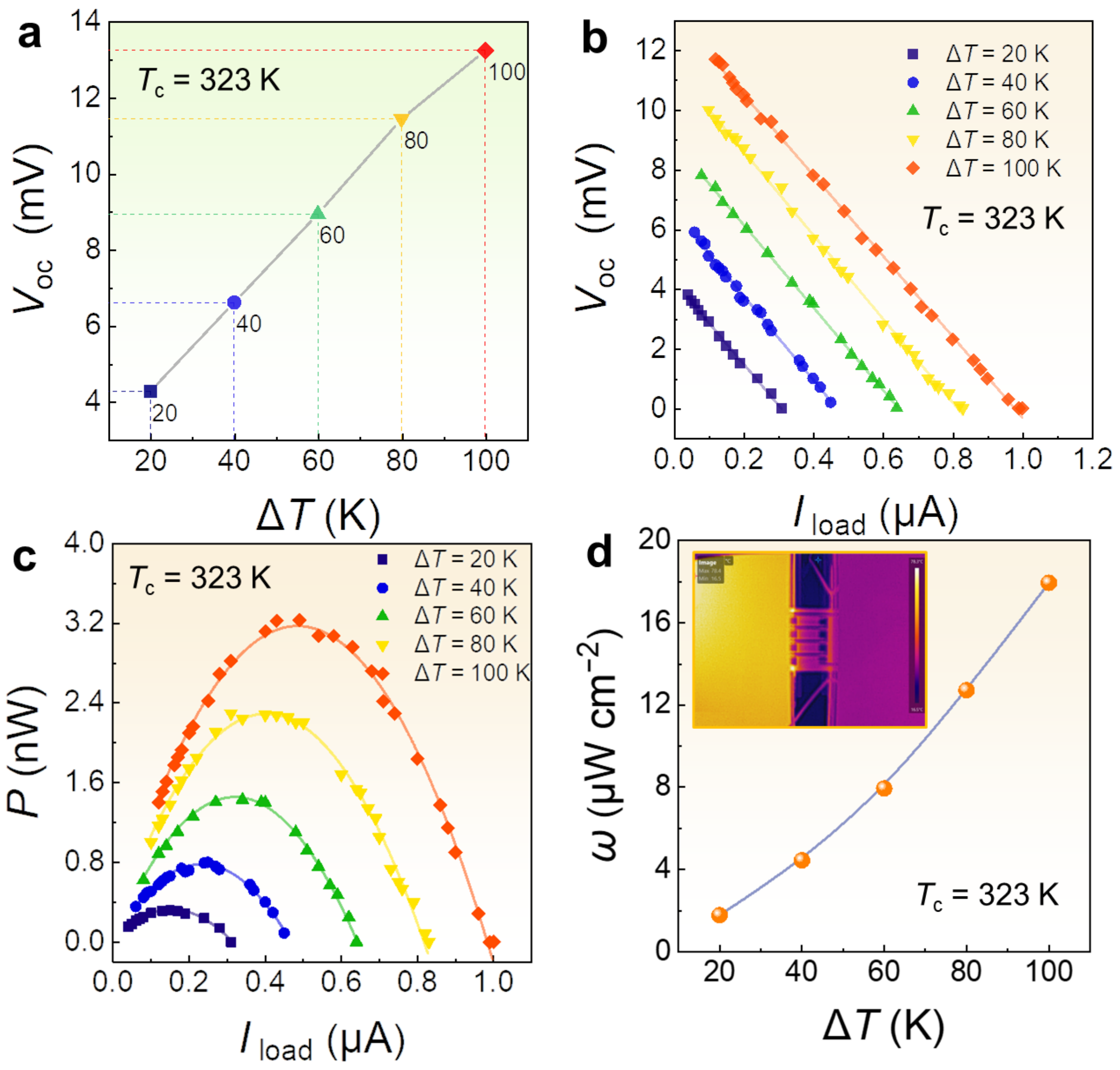

Supplementary Fig. 42 Output performance of film thermoelectric device when cold-side temperature T_c is 323 K. (**a**) Measured open-circuit voltages V_{oc} as a function of temperature difference ΔT for $Ce_{1.25}Fe_3CoSb_{12}$ flexible thin-film-based device. The cold-side temperature T_c is 323 K. The measured (**b**) V_{oc} and (**c**) output power P as a function of loading current I_{load} at different ΔTs. (**d**) Measured output power density ω as a function of ΔT. The inset shows the device during measurement.

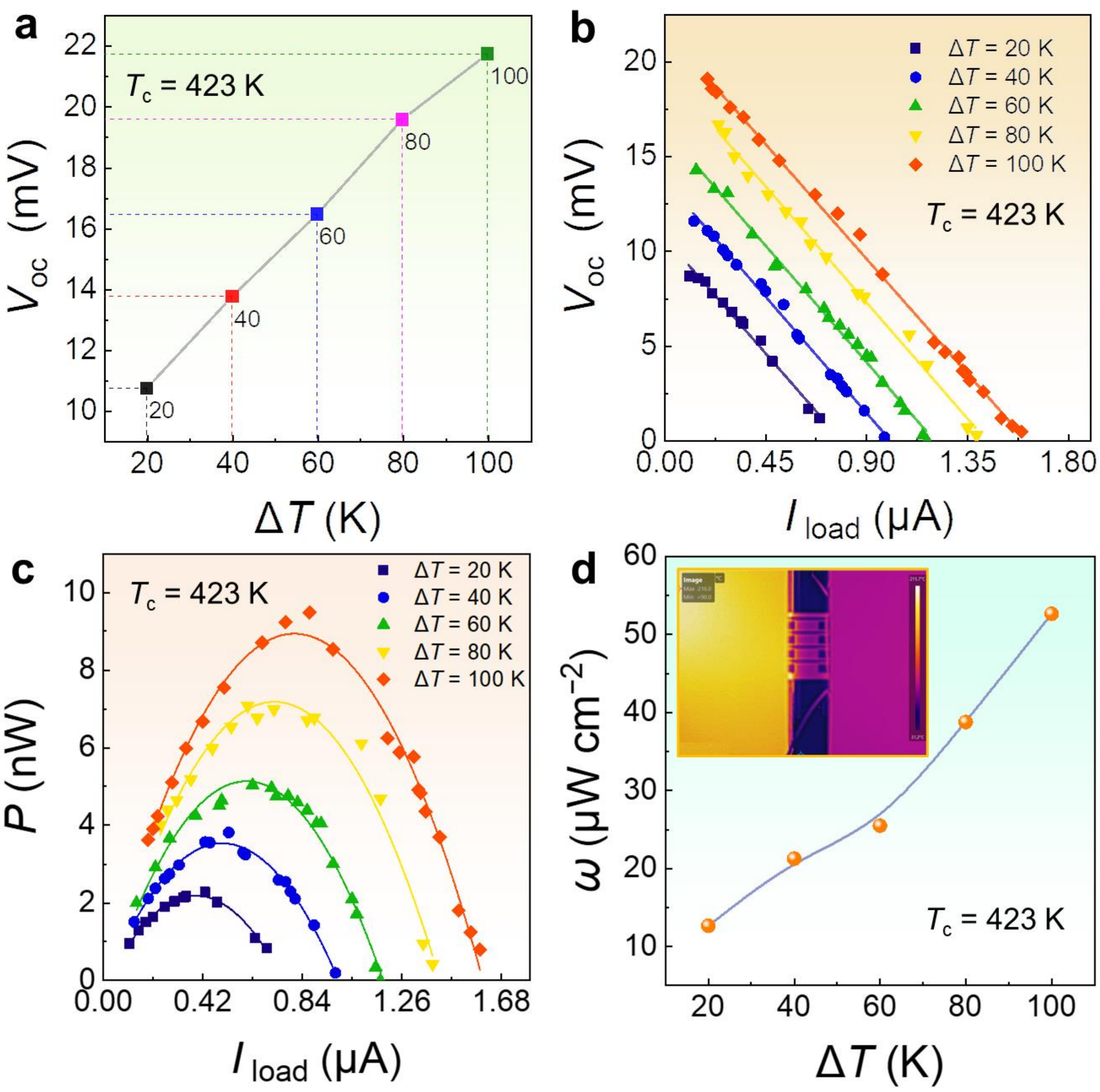

Supplementary Fig. 43 Output performance of film thermoelectric device when T_c is 423 K. (**a**) Measured V_{oc} as a function of ΔT for $Ce_{1.25}Fe_3CoSb_{12}$ flexible thin-film-based device. The T_c is 423 K. The measured (**b**) V_{oc} and (**c**) P as a function of I_{load} at different ΔTs. (**d**) Measured ω as a function of ΔT. The inset shows the device during measurement.

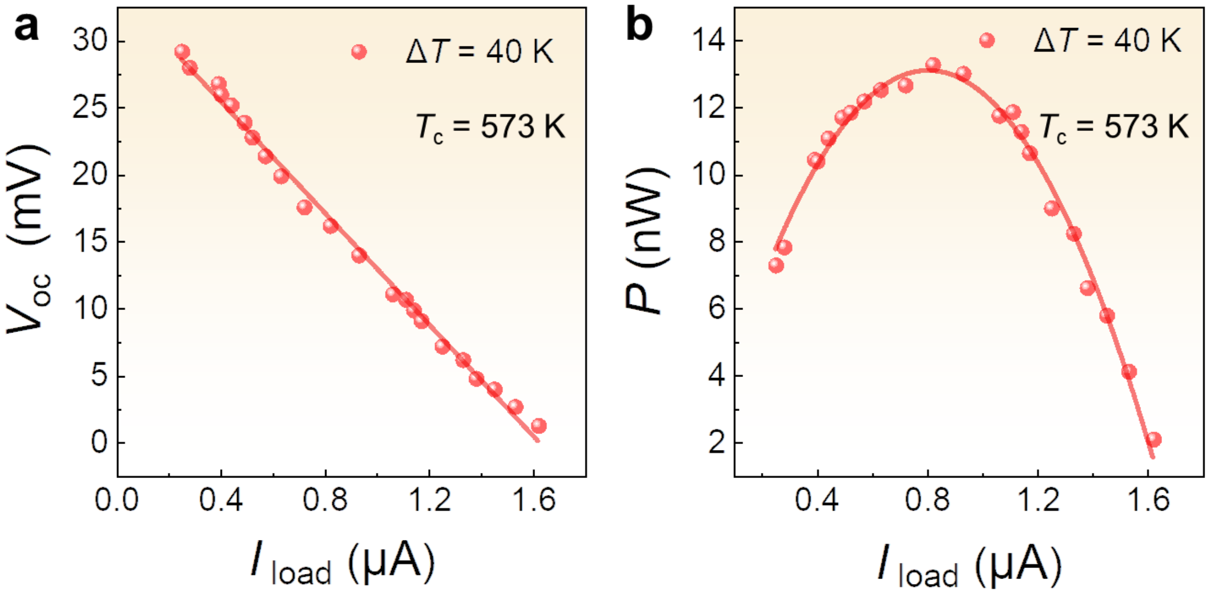

Supplementary Fig. 44. Output performance of film thermoelectric device when T_c is 523 K. The measured (**a**) V_{oc} and (**b**) P as a function of I_{load} at different ΔTs.

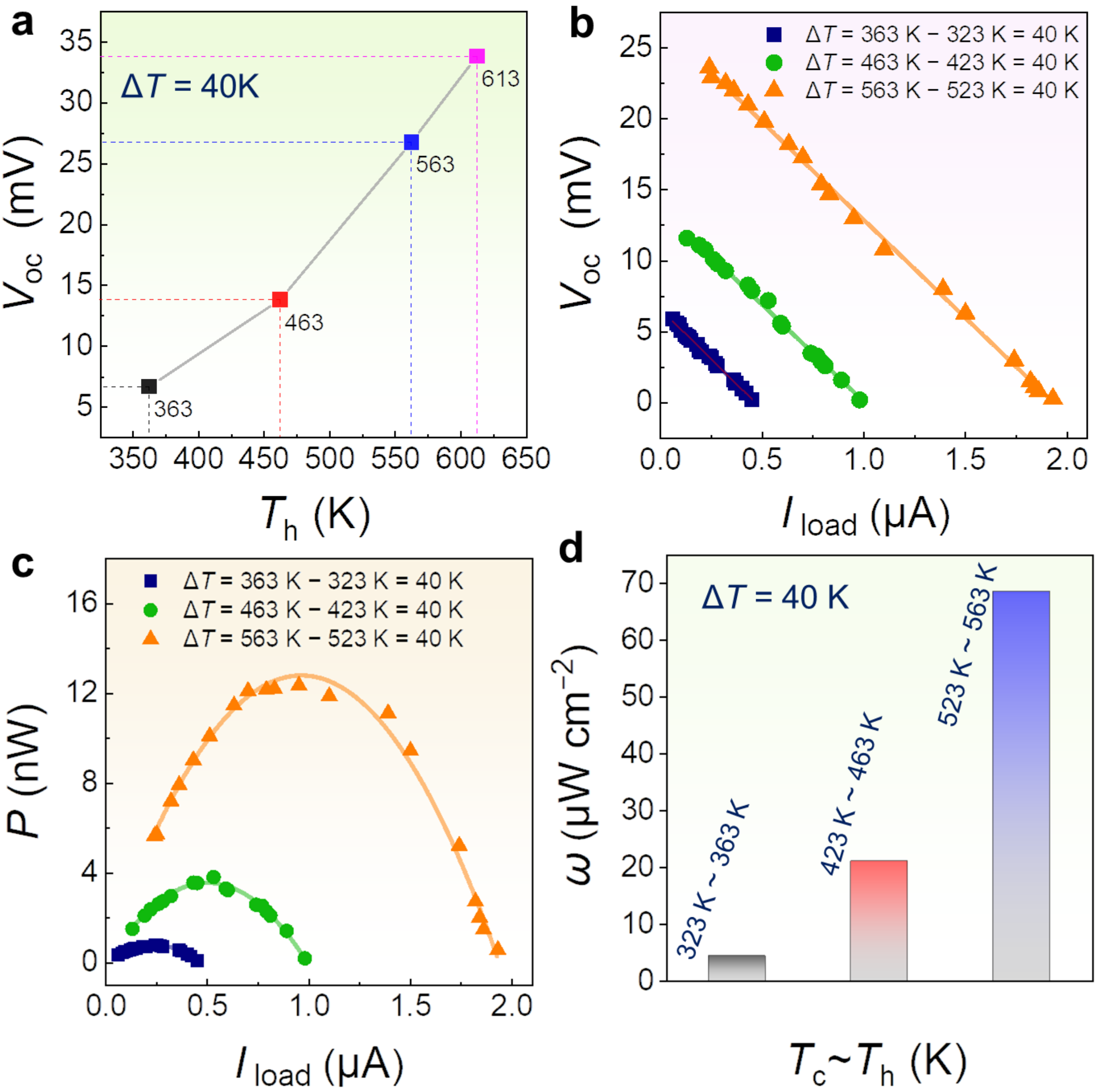

Supplementary Fig. 45 Output performance of film thermoelectric device at different cold-side and hot-side temperature. (**a**) Measured V_{oc} as a function of hot-side temperature T_h with same ΔTs of 40 K for $Ce_{1.25}Fe_3CoSb_{12}$ flexible thin-film-based device. The measured (**b**) V_{oc} and (**c**) P as a function of I_{load} at different T_h values with the same ΔTs of 40 K. (**d**) Measured ω as a function of T_h with the same ΔTs of 40 K.

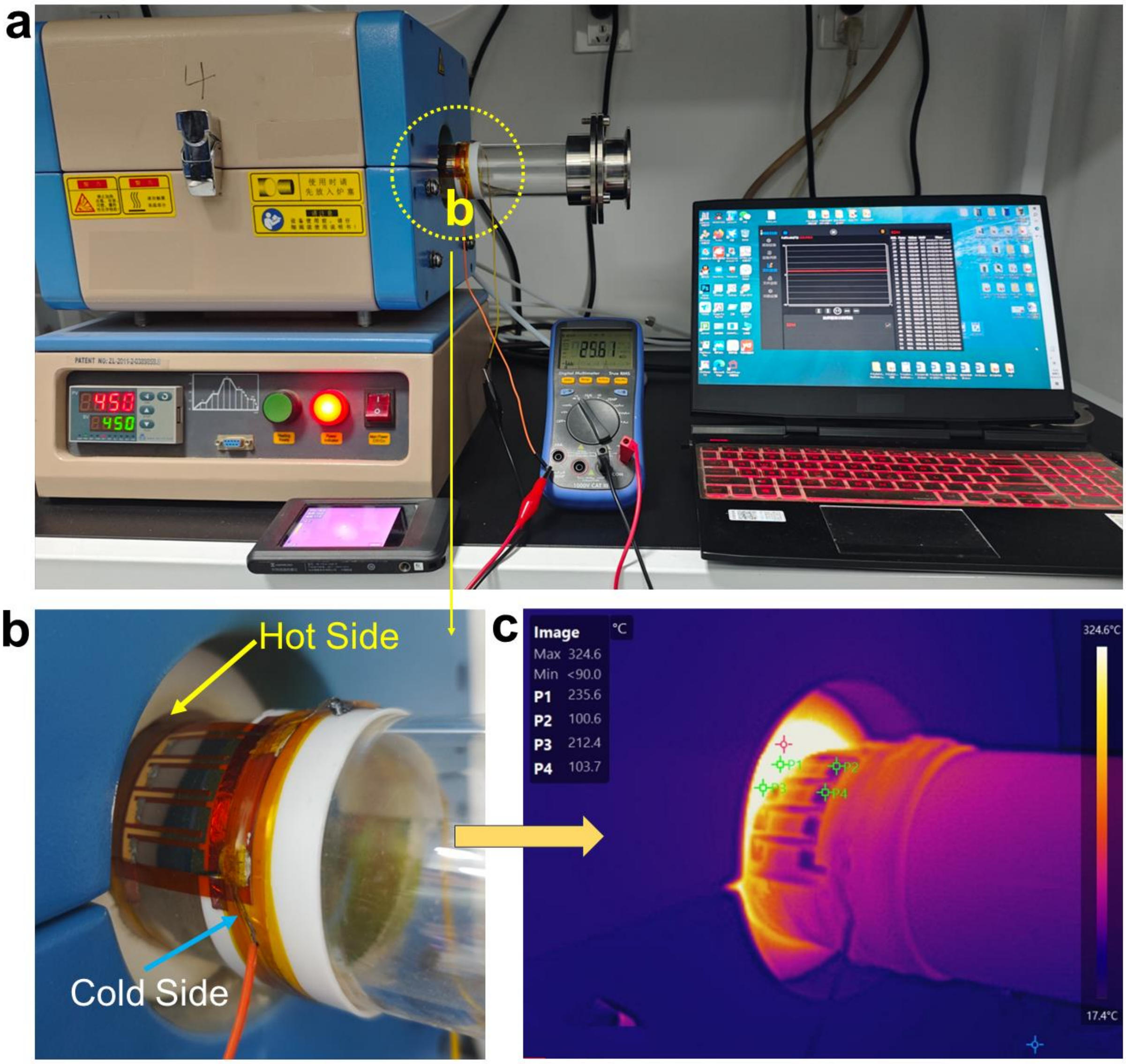

Supplementary Fig. 46 Photos that illustrate the high-temperature application of the as-fabricated device. (**a**) The $Ce_{1.25}Fe_3CoSb_{12}$ flexible thin-film-based device gathers residual heat from the curved surface of the tube furnace for power generation. (**b**) Image showing the device snugly affixed to the surface of a heating tube furnace. (**c**) Infrared image displaying the surface temperature distribution of the flexible thin-film-based device during waste heat power generation.

Supplementary Table 1. A summary of thermoelectric performance of p-type $CoSb_3$-based skutterudite thermoelectric thin films. Here *T* represents the absolute temperature, *σ* represents the electrical conductivity, *S* represents the Seebeck coefficient, $S^2\sigma$ represents the power factor, and *κ* represents the thermal conductivity. ITO is abbreviated from indium tin oxide.

**Inorganic**	**Substrate**	**Type**	***ZT***	***T* (K)**	***σ* (S cm^{-1})**	***S* (μV K^{-1})**	**$S^2\sigma$ (μW cm^{-1} K^{-2})**	***κ* (W m^{-1} K^{-1})**	**Flexible**	**Ref.**
**$Ce_{1.25}CoFe_3Sb_{12}$**	**Polyimide**	**p**	**0.58**	**653**	**105.1**	**98.1**	**1.01**	**0.11**	**Yes**	**This work**
**$CoSb_3$**	**Polyimide**	p	—	453	339.0	66	~1.5	—	Yes	17
**Fe-doped $CoSb_3$**	Silicon	p	—	420	108.6	254.0	~7.0	—	No	18
**Ti-doped $CoSb_3$**	BK7 glass	p	0.86	523	149.0	255.0	~9.70	0.62	No	19
**$CoSb_3$**	ITO	p	—	300	2100	58	~7.1	—	No	20
**$CoSb_3$**	Quartz	p	—	400	1818	26	~1.2	—	No	21
**$FeSb_3$**	SiO_2/Si	p	—	500	1400	100	~14	—	No	22
**$CoSb_3$**	Glass	p	—	713	77.4	154.5	~1.8	—	No	23
**$Sm_{0.3}(Fe_{0.63}Ni_{0.37})Sb_{12}$**	Silica	p	—	523	615	78	~3.7	—	No	24
**$LaFe_3CoSb_{12}$**	SiO_2/Si	p	—	400	769	68	~3.6	—	No	25
**$Ce_{0.1}Fe_{0.7}Ni_{3.29}Sb_{12}$**	quartz glass	p	—	470	158.7	139.2	~3.1	—	No	26
**$IrSb_3$**	silicon	p	—	298	—	303	—	—	No	27
**$Ce_{0.9}CoFe_3Sb_{12}$**	Al_2O_3	p	—	330	—	9.8	—	—	No	28

Supplementary Table 2. Detailed information of flexible polyimide substrate.

No.	Test content name		Unit	Tolerance range	Test result
**1**	Thickness		μm	125	125
**2**	Thickness tolerance range		μm	±5	—
**3**	Tensile strength	MD	MPa	≥135	139
		TD	MPa	≤115	118
**4**	Elongation	MD	%	≥35	55
		TD	%		70
**5**	Insulation strength	average	MV/m	≥135	135
		individual	MV/m	≥100	110
**6**	Surface resistivity (200℃)		Ω	$\geq 1.0*10^{13}$	$1.1*10^{13}$
**7**	Shrinkage (200℃ ± 5)	MD	‰	≤1.0	0.3
		TD		≤0.9	0.12
**8**	Breakdown voltage	thickness (125μm)	KV	≥5	5
**9**	Temperature resistance	thickness (125μm)	℃	350	350
**10**	Volume resistivity (200℃)		Ω m	$\geq 1.0*10^{13}$	$1.0*10^{13}$

Polyimide (PI) film test report. (Shenzhen Runsea Electronic Co.,)

Supplementary Table 3. A summary of the flexibility of flexible thermoelectric thin films. Here R/R_0 represents the normalized resistance and r represents the bending radius. CF is abbreviated from cellulose fiber.

**Circles**	$\boldsymbol{R/R_0}$	**Component**	***r*** **(mm)**	**Ref.**
1500	1.25	Ag_2Se	4	[29]
1000	1.11	CuAgSe	4	[30]
300	1.11	Bi_2Te_3 nanosheets	4	[31]
1000	1.10	Bi_2Te_3	4	[32]
100	1.04	Bi_2Te_3	10	[33]
2000	1.07	Sb_2Te_3/Te	6.5	[34]
2000	1.05	Bi_2Te_3	8	[35]
1000	1.14	Ag_2Se	/	[36]
1000	1.6	$CoSb_{3-x}Te_x$	4	[37]
1000	1.06	SnSe	4.5	[38]
1000	1. 2	$AgBi_{0.5}Sb_{1.5}Te_3$	5	[39]
1000	1.025	$Bi_{0.4}Sb_{1.6}Te_3$	10	[40]
1000	1.2	$Bi_{0.5}Sb_{1.5}Te_3$	5	[41]
2000	1.3	Bi_2Te_3	7	[42]
100	1.06	Bi_2Te_3-CF	10	[43]
600	1.04	Bi_2Te_3-CF	4	[44]
2000	1.06	$Ce_{1.25}Fe_3CoSb_{12}$	4	This work
1500	1.05	$Ce_{1.25}Fe_3CoSb_{12}$	4	This work
1000	1.03	$Ce_{1.25}Fe_3CoSb_{12}$	4	This work

Supplementary Table 4. EDS results for $Ce_{1.25}Fe_3CoSb_{12}$ film taken from Fig. 3a.

Region	Ce (at%)	Co (at%)	Fe (at%)	Sb (at%)
**1**	0.99	12.25	6.11	80.65
**2**	1.98	13.43	7.57	77.02
**3**	1.53	12.58	8.1	77.79
**4**	1.76	10.64	10.45	77.15
**5**	1.86	15.27	5.84	77.03
**6**	1.03	11.64	7.74	79.86
**7**	1.16	10.77	9.57	78.5
**8**	1.15	10.03	5.77	83.05
**9**	1.87	14.37	6.94	76.82
**10**	1.41	10.59	10.84	77.16
**11**	1.29	8.11	5.24	85.36
**12**	0.98	10.54	6.71	81.76
**13**	1.44	6.81	5.64	86.1
**14**	0.88	9.64	6.36	83.11

Supplementary Table 5. Comparison of nominal compositions and actual compositions for different thin films.

	**Composition**	**Ce(at%)**	**Co(at%)**	**Fe(at%)**	**Sb(at%)**
1	$Ce_{0.25}CoFe_3Sb_{12}$	1.54%	6.15%	18.46%	73.85%
	Actual component-1	0.10%	11.90%	7.50%	80.50%
2	$Ce_{0.50}CoFe_3Sb_{12}$	3.03%	6.06%	18.18%	72.73%
	Actual component-2	0.20%	21.90%	9.10%	70.10%
3	$Ce_{0.75}CoFe_3Sb_{12}$	4.48%	5.97%	17.91%	71.64%
	Actual component-3	0.20%	21.90%	12.00%	66.00%
4	$Ce_{1.25}CoFe_3Sb_{12}$	7.25%	5.80%	17.39%	69.57%
	Actual component-4	0.30%	21.20%	8.70%	69.70%
5	$Ce_{1.50}CoFe_3Sb_{12}$	8.57%	5.71%	17.14%	68.57%
	Actual component-5	0.20%	22.70%	11.20%	65.90%

References

1. Kresse, G. & Hafner, J. *Ab initio* molecular-dynamics simulation of the liquid-metal-amorphous-semiconductor transition in germanium. *Phys. Rev. B* **49**, 14251-14269 (1994).
2. Kresse, G. & Hafner, J. *Ab initio* molecular dynamics for liquid metals. *Phys. Rev. B* **47**, 558-561 (1993).
3. Kresse, G. & Furthmüller, J. Efficiency of *ab-initio* total energy calculations for metals and semiconductors using a plane-wave basis set. *Comp. Mater. Sci.* **6**, 15-50 (1996).
4. Kresse, G. & Hafner, J. Norm-conserving and ultrasoft pseudopotentials for first-row and transition elements. *J. Phys. Condens. Mat.* **6**, 8245-8257 (1994).
5. Kresse, G. & Furthmüller, J. Efficient iterative schemes for *ab initio* total-energy calculations using a plane-wave basis set. *Phys. Rev. B* **54**, 11169-11186 (1996).
6. Kresse, G. & Joubert, D. From ultrasoft pseudopotentials to the projector augmented-wave method. *Phys. Rev. B* **59**, 1758-1775 (1999).
7. Perdew, J.P., Burke, K. & Ernzerhof, M. Generalized gradient approximation made simple. *Phys. Rev. Lett.* **77**, 3865-3868 (1996).
8. Setyawan, W. & Curtarolo, S. High-throughput electronic band structure calculations: challenges and tools. *Comp. Mater. Sci.* **49**, 299-312 (2010).
9. Shi, X.L. et al. Boosting the thermoelectric performance of p-type heavily Cu-doped polycrystalline SnSe *via* inducing intensive crystal imperfections and defect phonon scattering. *Chem. Sci.* **9**, 7376-7389 (2018).
10. Shi, X. et al. High thermoelectric performance in p-type polycrystalline Cd-doped SnSe achieved by a combination of cation vacancies and localized lattice engineering. *Adv. Energy Mater.* **9**, 1803242 (2019).
11. Shi, X. et al. Polycrystalline SnSe with extraordinary thermoelectric property *via* nanoporous design. *ACS Nano* **12**, 11417-11425 (2018).

12. Jin, M. et al. Super large $Sn_{1-x}Se$ single crystals with excellent thermoelectric performance. *ACS Appl. Mater. Interfaces* **11**, 8051-8059 (2019).

13. Caillat, T., Kulleck, J., Borshchevsky, A. & Fleurial, J.P. Preparation and thermoelectric properties of the skutterudite-related phase $Ru_{0.5}Pd_{0.5}Sb_3$. *J. Appl. Phys.* **79**, 8419-8426 (1996).

14. Caillat, T., Borshchevsky, A. & Fleurial, J.P. Properties of single crystalline semiconducting $CoSb_3$. *J. Appl. Phys.* **80**, 4442-4449 (1996).

15. Sales, B.C., Mandrus, D., Chakoumakos, B.C., Keppens, V. & Thompson, J.R. Filled skutterudite antimonides: Electron crystals and phonon glasses. *Phys. Rev. B* **56**, 15081-15089 (1997).

16. Wang, H., Li, S., Li, X. & Zhong, H. Microstructure and thermoelectric properties of doped p-type $CoSb_3$ under TGZM effect. *J. Cryst. Growth* **466**, 56-63 (2017).

17. Zheng, Z.-h. et al. Thermoelectric properties and micro-structure characteristics of nano-sized $CoSb_3$ thin films prefabricating by co-sputtering. *J. Alloys Compd.* **732**, 958-962 (2018).

18. Masarrat, A. et al. Effect of Fe ion implantation on the thermoelectric properties and electronic structures of $CoSb_3$ thin films. *RSC Adv.* **9**, 36113-36122 (2019).

19. Liang, G.-x. et al. Nano structure Ti-doped skutterudite $CoSb_3$ thin films through layer inter-diffusion for enhanced thermoelectric properties. *J. Eur. Ceram. Soc.* **39**, 4842-4849 (2019).

20. Yadav, S., Yadav, B.S., Chaudhary, S. & Pandya, D.K. Deposition potential controlled structural and thermoelectric behavior of electrodeposited $CoSb_3$ thin films. *RSC Adv.* **7**, 20336-20344 (2017).

21. Bala, M. et al. Evolution of nanostructured single-phase $CoSb_3$ thin films by low-energy ion beam induced mixing and their thermoelectric performance. *Phys. Chem. Chem. Phys.* **19**, 24886-24895 (2017).

22. Daniel, M.V. et al. Structural and thermoelectric properties of $FeSb_3$ skutterudite thin films. *Phys. Rev. B* **91**, 085410 (2015).

23. Yang, Y.J. et al. Thermoelectric properties of $CoSb_3$ nanoparticle films. *Adv. Mater. Res.* **347-353**, 3448-3455 (2012).

24. Latronico, G. et al. Effect of the annealing treatment on structural and transport properties of thermoelectric $Sm_y(Fe_xNi_{1-x})_4Sb_{12}$ thin films. *Nanotechnology* **34**, 115705 (2023).

25. Suzuki, A. Pulsed laser deposition of filled skutterudite $LaFe_3CoSb_{12}$ thin films. *Jpn. J. Appl. Phys.* **42**, 2843 (2003).

26. Zeipl, R. et al. Properties of thin n-type $Yb_{0.14}Co_4Sb_{12}$ and p-type $Ce_{0.09}Fe_{0.67}Co_{3.33}Sb_{12}$ skutterudite layers prepared by laser ablation. *J. Vac. Sci. Technol. A* **28**, 523-527 (2010).

27. Caylor, J.C., Stacy, A.M., Gronsky, R. & Sands, T. Pulsed laser deposition of skutterudite thin films. *J. Appl. Phys.* **89**, 3508-3513 (2001).

28. Arnache, O. et al. Electrical and structural properties of $Ce_{0.9}CoFe_3Sb_{12}$ thermoelectric thin films. *Solid State Commun.* **133**, 343-346 (2005).

29. Ding, Y. et al. High performance n-type Ag_2Se film on nylon membrane for flexible thermoelectric power generator. *Nat. Commun.* **10**, 841 (2019).

30. Lu, Y. et al. Ultrahigh power factor and flexible silver selenide-based composite film for thermoelectric devices. *Energy Environ. Sci.* **13**, 1240-1249 (2020).

31. Chen, X. et al. Flexible thermoelectric films based on Bi_2Te_3 nanosheets and carbon nanotube network with high n-type performance. *ACS Appl. Mater. Interfaces* **13**, 5451-5459 (2021).

32. Lu, Y. et al. Staggered-layer-boosted flexible Bi_2Te_3 films with high thermoelectric performance. *Nat. Nanotechnol.* **18**, 1281-1288 (2023).

33. Jin, Q. et al. Flexible layer-structured Bi_2Te_3 thermoelectric on a carbon nanotube scaffold. *Nat. Mater.* **18**, 62-68 (2019).

34. Wei, M. et al. Directional thermal diffusion realizing inorganic Sb_2Te_3/Te hybrid thin films with high thermoelectric performance and flexibility. *Adv. Funct. Mater.* **32**, 2207903 (2022).

35. Zheng, Z.-H. et al. Harvesting waste heat with flexible Bi_2Te_3 thermoelectric thin film. *Nat. Sustain.* **6**, 180-191 (2023).

36. Zang, J. et al. Effect of post-annealing treatment on the thermoelectric properties of Ag_2Se flexible thin film prepared by magnetron sputtering method. *Res. in Phys.* **45**, 106222 (2023).

37. Kato, A. et al. Facile fabrication of n-type flexible $CoSb_{3-x}Te_x$ skutterudite/PEDOT:PSS hybrid thermoelectric films. *Polymers* **14**, 1986 (2022).

38. Rongione, N.A. et al. High-performance solution-processable flexible SnSe nanosheet films for lower grade waste heat recovery. *Adv. Electron. Mater.* **5**, 1800774 (2019).

39. Shang, H. et al. High-performance Ag-modified $Bi_{0.5}Sb_{1.5}Te_3$ films for the flexible thermoelectric generator. *ACS Appl. Mater. Interfaces* **12**, 7358-7365 (2020).

40. Varghese, T. et al. Flexible thermoelectric devices of ultrahigh power factor by scalable printing and interface engineering. *Adv. Funct. Mater.* **30**, 1905796 (2020).

41. Shang, H. et al. $Bi_{0.5}Sb_{1.5}Te_3$-based films for flexible thermoelectric devices. *J. Mater. Chem. A* **8**, 4552-4561 (2020).

42. Kong, D., Zhu, W., Guo, Z. & Deng, Y. High-performance flexible Bi_2Te_3 films based wearable thermoelectric generator for energy harvesting. *Energy* **175**, 292-299 (2019).

43. Jin, Q. et al. Cellulose fiber-based hierarchical porous bismuth telluride for high-performance flexible and tailorable thermoelectrics. *ACS Appl. Mater. Interfaces* **10**, 1743-1751 (2018).

44. Komatsu, N. et al. Macroscopic weavable fibers of carbon nanotubes with giant thermoelectric power factor. *Nat. Commun.* **12**, 4931 (2021).
