# Supplementary material for: High-performance flexible p-type Ce-filled Fe3CoSb12 skutterudite thin film for medium-to-high-temperature applications
Source: Nat Commun. 2024 May 18;15:4242. doi: 10.1038/s41467-024-48677-4 (PMC11102547; doi:10.1038/s41467-024-48677-4)
Supplement: Supplementary file 3 — Description of Additional Supplementary Files [file 41467_2024_48677_MOESM3_ESM.pdf]

Description of Additional Supplementary Files

Supplementary Movie 1:

Bending flexible films with a homemade automatic bending instrument.

Supplementary Movie 2:

Bending flexible films with a homemade automatic bending instrument.

Supplementary Movie 3:

Detailed information on each component of the platform used for temperature detection.

Supplementary Movie 4:

The variation of detected voltage V signal with temperature.

Supplementary Movie 5:

The variation of detected current I signal with temperature.

Supplementary Movie 6:

Measuring thermoelectric power generation in $CoSb_3$ thin-film-based thermoelectric devices.

Supplementary Movie 7:

Collecting waste heat for power generation in a scenario involving a curved high-temperature surface.
